# Transcriptome and Secretome Analysis of Intra-Mammalian Life-Stages of *Calicophoron daubneyi* Reveals Adaptation to a Unique Host Environment

**DOI:** 10.1074/mcp.RA120.002175

**Published:** 2021-02-11

**Authors:** Kathryn M. Huson, Erwan Atcheson, Nicola A.M. Oliver, Philip Best, Jason P. Barley, Robert E.B. Hanna, Tom N. McNeilly, Yongxiang Fang, Sam Haldenby, Steve Paterson, Mark W. Robinson

**Affiliations:** 1 School of Biological Sciences, Queen’s University Belfast, Belfast, Northern Ireland; 2 Veterinary Sciences Division, Agri-Food and Biosciences Institute, Belfast, Northern Ireland; 3 Disease Control Department, Moredun Research Institute, Edinburgh, Scotland; 4 Centre for Genomic Research, University of Liverpool, Liverpool, England

**Keywords:** *Calicophoron daubneyi*, rumen fluke, paramphistome, secretome, transcriptome, diagnostic, coproantigen, ELISA, AbD, antibody diluent, DE, differential expression, E/S, excretory/secretory, emPAI, exponentially modified protein abundance index, FA, formic acid, FaBP, fatty acid–binding protein, HDM, helminth defense molecule, NEJ, newly excysted juvenile, TCA, tricarboxylic acid, TPM, transcripts per million, α-CdE/S, anti–*C. daubneyi* excretory/secretory protein antibody

## Abstract

Paramphistomosis, caused by the rumen fluke, *Calicophoron daubneyi*, is a parasitic infection of ruminant livestock, which has seen a rapid rise in prevalence throughout Western Europe in recent years. After ingestion of metacercariae (parasite cysts) by the mammalian host, newly excysted juveniles (NEJs) emerge and invade the duodenal submucosa, which causes significant pathology in heavy infections. The immature flukes then migrate upward, along the gastrointestinal tract, and enter the rumen where they mature and begin to produce eggs. Despite their emergence, and sporadic outbreaks of acute disease, we know little about the molecular mechanisms used by *C. daubneyi* to establish infection, acquire nutrients, and avoid the host immune response. Here, transcriptome analysis of four intramammalian life-cycle stages, integrated with secretome analysis of the NEJ and adult parasites (responsible for acute and chronic diseases, respectively), revealed how the expression and secretion of selected families of virulence factors and immunomodulators are regulated in accordance with fluke development and migration. Our data show that while a family of cathepsins B with varying S2 subsite residues (indicating distinct substrate specificities) is differentially secreted by NEJs and adult flukes, cathepsins L and F are secreted in low abundance by NEJs only. We found that *C. daubneyi* has an expanded family of aspartic peptidases, which is upregulated in adult worms, although they are under-represented in the secretome. The most abundant proteins in adult fluke secretions were helminth defense molecules that likely establish an immune environment permissive to fluke survival and/or neutralize pathogen-associated molecular patterns such as bacterial lipopolysaccharide in the microbiome-rich rumen. The distinct collection of molecules secreted by *C. daubneyi* allowed the development of the first coproantigen-based ELISA for paramphistomosis which, importantly, did not recognize antigens from other helminths commonly found as coinfections with rumen fluke.

## Highlights

• The first transcriptome data for intramammalian stages of *Calicophoron daubneyi.*
• Comparative secretome profiles of infective stage and adult flukes.
• New mechanistic insights into infectivity and pathogenicity.
• Development of the first coproantigen-based ELISA for paramphistomosis.

## Graphical abstract

**Figure:**
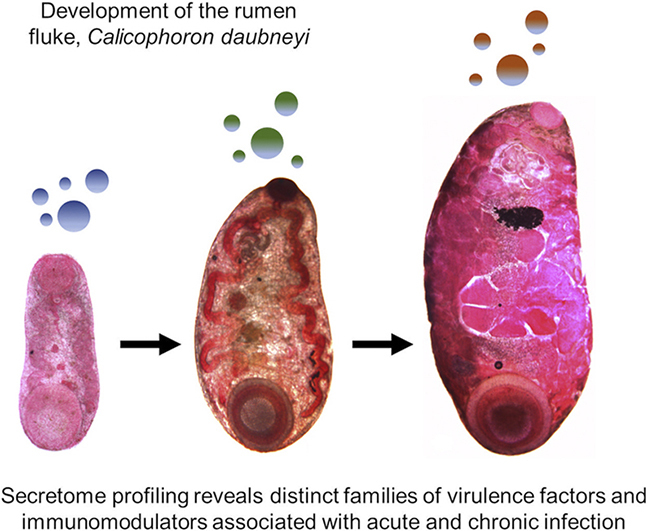

## In brief

Paramphistomosis, caused by *Calicophoron daubneyi*, is a parasitic infection of ruminant livestock currently spreading throughout Western Europe. Here, transcriptome analysis of four intramammalian life-cycle stages, integrated with secretome profiling of the infective stage and adult parasites (responsible for acute and chronic diseases, respectively), revealed how selected families of unique virulence factors and immunomodulators are regulated in accordance with fluke development and migration. This analysis allowed the development of the first coproantigen-based ELISA for paramphistomosis.

Infections by parasitic fluke are an important animal health and production concern for livestock producers worldwide. In the United Kingdom, and throughout Europe, the liver fluke (*Fasciola hepatica*) has historically been a major focus for producers. In recent years, however, there has been a sharp increase in both the incidence and severity of rumen fluke (the paramphistome *Calicophoron daubneyi*) infections in both sheep and cattle (1). Paramphistomes are endemic to subtropical and tropical regions and are believed to have been carried into Western Europe from North Africa *via* the movement of ruminant livestock (2). Although the exact reasons for the rise in rumen fluke infections in Europe are not fully understood, the increase in warm wet summers and mild winters—conditions that favor *Galba truncatula*, the snail intermediate host of *C. daubneyi*—is thought to be a major contributing factor (3).

Acute, clinical paramphistomosis is caused when grazing stock ingest large numbers of metacercariae from pasture, which then excyst *en masse* in the duodenum (4). The newly excysted juvenile (NEJ) flukes then migrate into the intestinal submucosa causing significant damage to the host tissue (5). Large areas of damaged small intestine may hemorrhage, causing significant blood loss and hypoalbuminemia, frequently resulting in mortality at this point (6, 7). After a period spent feeding on the host tissue in the small intestine, immature paramphistomes migrate to the rumen where they mature, and infections become patent (8). Although chronic infections are generally seen as well tolerated, postmortem observations have noted both rumenitis and abomasitis in infected animals (9, 10), along with atrophy of the rumen papillae (1, 10).

Despite a prevalence of 55% to 77% (1), clinical disease is still relatively rare in the United Kingdom/Ireland. However, fatal disease outbreaks, linked to significant immature parasite burdens, have been reported in both sheep and cattle in recent years (4, 6, 10, 11, 12, 13). Control of fluke infection currently relies on anthelmintic drugs. While several drugs show efficacy against liver fluke, only one anthelmintic (oxyclozanide) is effective against rumen fluke (14, 15). This makes correct diagnosis imperative. However, detection of rumen fluke infection currently requires either examination of animals at postmortem or labor-intensive fecal egg counts that only detect chronic infection due to the presence of egg-producing adult flukes. Thus, the development of new tools for more rapid diagnosis of *C. daubneyi* (including acute infection) is required.

Compared with other helminths of veterinary importance, *C. daubneyi* remains a poorly studied species. We have much to learn about its basic biology and interactions with the ruminant host, particularly the infective juvenile stages (1). Here, we have performed the first transcriptomic analysis of all four major intramammalian life-cycle stages of *C. daubneyi*. These data, coupled with secretome analysis of the infective and adult stages that are associated with acute and chronic diseases, respectively, have provided a greater understanding of the key biochemical and molecular mechanisms underpinning *C. daubneyi* infectivity, migration, and development within its ruminant host. Our data reveal how the parasite regulates expression and secretion of a collection of molecules required for tissue invasion, nutrition, and modulation of the host immune response according to fluke development and exposure to different host microenvironments (*i.e.*, the duodenum and rumen). Owing to the unique composition of *C. daubneyi* secretions compared with those of *F. hepatica* (often found as coinfections), the diagnostic potential of these molecules was investigated. Accordingly, we present the first ELISA-based assay capable of detecting *C. daubneyi* antigens in fecal samples from naturally infected cattle. Our data represent an important foundation for future studies aimed at understanding rumen fluke infectivity and the development of future treatment options and diagnostic tests.

## Experimental Procedures

### Experimental Design and Statistical Rationale

For transcriptomics studies, 3 biological replicates of each life-cycle stage were prepared for Illumina RNA-Seq, with 1 replicate used for PacBio sequencing for use as full-length transcript scaffold data during assembly. Three biological replicates were also used for the proteomics study of excretory/secretory (E/S) products analyzed by LC-MS/MS. ELISA data are representative of three independent experiments.

### Source of Parasite Material and RNA Extraction

To obtain RNA samples, *C. daubneyi* adults and newly migrated parasites were collected from the rumen of a naturally-infected cow in a local abattoir (Dungannon, Northern Ireland). Individual adults and small groups (approx. 6) of newly migrated flukes were rinsed briefly in sterile warm (39 °C) PBS and immediately placed into TRIzol reagent (Life Technologies) to preserve mRNA. Immature parasites were removed from the duodenum of a recently deceased bovine host during postmortem examination, and approximately 200 mg of parasite material placed directly into TRIzol reagent. Rumen fluke metacercariae (Ridgeway Research) were excysted *in vitro* and the resulting NEJ parasites cultured in RPMI-1640 medium for 24 h as described previously (16). The culture media was removed, and TRIzol reagent was added directly to groups of 500 NEJs. All samples were stored in TRIzol at −80 °C until RNA extraction. Samples were homogenized in TRIzol, and total RNA was extracted following the manufacturer’s protocol. RNA yields were quantified using a NanoDrop spectrophotometer and shipped to the Centre for Genomic Research (University of Liverpool, UK) for library construction. Before sequencing, the rumen fluke species was confirmed by PCR using *C. daubneyi*–specific primers targeting an 885-bp region of the cytochrome oxidase 1 gene; Cd_Cox1F 5’-TGGAGAGTTTGGCGTCTTTT-3’ and Cd_Cox1R 5’-CCATCTTCCACCTCATCTGG-3’ as previously described (17).

### PacBio Iso-Seq Library Preparation and Sequencing

Full-length cDNA was made from 1 μg of total RNA from each sample using the TeloPrime full-length cDNA amplification kit from Lexogen. The final second strand product was amplified for 20 cycles and converted to a PacBio SMRTbell library using the SMRTbell Template Prep Kit 1.0. Each sample was run on a single Sequel SMRT cell v2 using MagBead loading and 10-h movie times. PacBio reads were analyzed using the Iso-seq pipeline (SMRT Link version 4.0.0.190159), with default parameters. Each sample was processed independently. Subsequently, high- and low-quality full-length isoforms from each sample were clustered using CD-HIT (18). Cluster representatives were polished with Arrow (19) and reclustered with cd-hit-est, as above but with more relaxed parameters to limit overclustering (-aL 0.97). These two steps were iteratively performed to generate a set of 27,949 isoforms, which were subsequently filtered to remove any sequences that (a) had a mean base quality score of less than 50 or (b) had more than 1% of bases with a quality score below 10. This yielded a final set of 27,254 isoforms.

### Annotation of Transcripts

Putative proteins sequences were obtained for isoforms using TransDecoder (version 3.0.1; (20)), selecting for a minimum amino acid sequence length of 50. Sequences were annotated with input for the Trinotate pipeline (version 3.1.0; (20)), according to the proposed guidelines from the authors. The BLAST (21) was used to align nucleotide and protein sequences against UniProt (release 2017_11) and TrEMBL and against proteins encoded by *F. hepatica* gene models available at WormBase ParaSite (ftp://ftp.ebi.ac.uk/pub/databases/wormbase/parasite/releases/WBPS2/species/fasciola_hepatica/PRJEB6687/fasciola_hepatica.PRJEB6687.WBPS2.protein.fa.gz). All BLAST alignments which yielded e-values of 1 x 10^-5^ or lower were reported. Predicted amino acid sequences were compared with Pfam-A (release 31.0) (22), using hmmscan (version 3.1b1; (23)). Signal peptides and transmembrane domains were predicted using SignalP (version 4.1; (24)) and TMHMM (version 2.0; (25)).

### Illumina RNA-Seq Library Preparation and Sequencing

Triplicate total RNA samples (from *C. daubneyi* NEJs, immature intestinal flukes, newly migrated flukes, and mature adults) were treated with Turbo DNase (Life Technologies) to remove all contaminating DNA. 1 μg total RNA was selected for poly A using NEBNext Poly(A) mRNA Magnetic Isolation Module. RNA-Seq libraries were prepared from the enriched material using the NEBNext Ultra Directional RNA Library Prep Kit for Illumina using 12 cycles of amplification. Final libraries were pooled in equimolar amounts using the Qubit and Bioanalyzer data and checked by quantitative PCR for template loading calculations. DNA was diluted to a loading concentration of 300 pM. The RNA libraries were sequenced on an Illumina HiSeq 4000 platform with version 1 chemistry using sequencing by synthesis (SBS) technology to generate 2 x 150 bp paired-end reads.

### RNA-Seq Data Processing and Quality Filtering

Base calling and de-multiplexing of indexed reads was performed by CASAVA version 1.8.2 (Illumina). Sequences were trimmed using Cutadapt, version 1.2.1 (26), to remove any adaptor sequences matching with 3 or more bases. The reads were further trimmed to remove low-quality bases, using Sickle, version 1.200, with a minimum window quality score of 20. Illumina reads were aligned to the final Iso-Seq isoforms using Bowtie2, version 2.1.0 (27), accepting all global concordantly paired alignments.

### Differential Expression Analysis

Read alignment data were used as input to BitSeq (28), to estimate raw expression values for each transcript in the dataset. Differential transcript expression analysis was applied to the read count data, which was conducted in the R environment, using the DESeq2 package (29). Transcripts per million (TPM) values were calculated by dividing the counts to each transcript by the length of the transcript and then normalized further to generate counts per million reads. Transcripts were clustered according to their log-fold changes in expression between sample groups, using k-means clustering functions in R.

### Discovery and Characterization of *C. daubneyi* Gene Families

*C. daubneyi* gene families and specific homologs of interest were identified using BlastP (1.0 x 10^-6^ e-value cut-off) run locally with BioEdit (30) using published query sequences from other trematode species (*F. hepatica, Fasciola gigantica, Opisthorchis viverrini, Clonorchis sinensis,* and *Schistosoma* spp.). Duplicate and truncated sequences were manually removed from the BLAST hits, and transcript TPM values were extracted for the remaining transcripts at each of the 4 life-cycle stages to allow differential expression (DE) analysis across the intramammalian stages of the *C. daubneyi* lifecycle and used to produce heatmaps *via* the heatmapper.ca expression tool (31). Helical wheel analysis was conducted on *C. daubneyi* HDM family members (http://tcdb.org/progs/helical_wheel.php) to identify amphipathic regions.

Neighbor-joining phylogenetic trees were constructed for selected protein sequences using MEGA6 (32). The bootstrap consensus tree inferred from 1000 replicates is taken to represent the evolutionary history of the taxa analyzed (33). Branches corresponding to partitions reproduced in less than 50% bootstrap replicates are collapsed. The percentage of replicate trees in which the associated taxa clustered together in the bootstrap test (1000 replicates) is shown next to the branches. The evolutionary distances were computed using the Poisson correction method (34) and are in the units of the number of amino acid substitutions per site.

### Collection of Parasite E/S Products

To prepare secretions, adult rumen flukes were thoroughly washed (3 x 5 min) with warm (39 °C) sterile PBS to void their gut contents and to remove any host contaminants. The integrity and movement of the flukes were monitored by microscopy and any found to be damaged or dead were discarded before culture. Flukes were then maintained in RPMI-1640 culture medium containing 0.1% glucose, 100 U penicillin, and 100 μg/ml streptomycin (Sigma-Aldrich), at 1 worm/ml for 5 h at 39 °C. Whole E/S products were also collected from *C. daubneyi* NEJs (in groups of 500 parasites) as described previously (16). Excysted NEJs were cultured in 1-ml RPMI-1640 supplemented with 100 IU mL^−1^ penicillin and 100-mg mL^−1^ streptomycin for 24 h at 39 °C. The culture media was recovered and centrifuged for 30 min at 1500*g* to remove large debris. Adult and NEJ E/S fractions were concentrated (approx. 10-fold) using Amicon Ultra Centrifugal Filter Units with a 3-kDa molecular weight cutoff (Millipore, UK) and stored at –80 °C until further analysis.

E/S proteins were reduced with 2-mM DTT in 50-mM NH_4_HCO_3_ at 60 °C for 20 min and alkylated with 5-mM iodoacetamide at room temperature (RT) (18–21 °C) in the dark for 30 min. E/S samples were incubated with 100 ng/μl sequencing grade trypsin (Promega) overnight at 37 °C. The digestions were stopped by the addition of TFA to a final concentration of 0.1%.

### MS analysis of *C. daubneyi* Proteins

E/S proteins were analyzed in biological triplicate. Tryptic peptides were dried in a vacuum centrifuge and reconstituted with 10 μl of 0.1% TFA before analysis by LC-MS/MS. Peptides is 5 μl of the resulting suspension were purified using an Acclaim PepMap 100 column (C18, 100 μM x 2 cm) before delivery to an analytical column (Eksigent C18-CL NanoLC Column, 3 μm; 75 μm x 15 cm) equilibrated in 5% acetonitrile/0.1% formic acid (FA). Elution was carried out with a linear gradient of 5 to 35% buffer B in buffer A for 30 min (buffer A: 0.1% FA; buffer B: acetonitrile, 0.1% FA) at a flow rate of 300 nl/min. Peptides were analyzed using an LTQ OrbiTrap Velos Pro (Thermo Scientific) operating in an information-dependent acquisition mode using a top 15 method. MS spectra were acquired in the Orbitrap analyzer with a mass range of 335 to 1800 m/z, with a resolution of 60,000 in the Orbitrap. Collision-induced dissociation peptide fragments were acquired in the ion trap with a collision energy of 35, activation energy of 0.25, and 10-ms activation time, with a default charge state of 2 for fragment ions. Orbitrap Velos RAW data files were extracted and converted to Mascot generic files (.mgf) for database searching using Mascot v2.4.1 (Matrix Science, London, UK).

### Database Searching

All MS/MS samples were analyzed using Mascot (version 2.4.1, Matrix Science, London, UK). Mascot was set up to search the LIMS12524_20171212 *C. daubneyi* transcriptome database (version 1.0, 48,899 entries) assuming the digestion enzyme strict trypsin with 1 missed cleavage allowed. The transcript data can be accessed *via* the European Nucleotide Archive (www.ebi.ac.uk/ena) under accession number PRJEB28150. Mascot was searched with a fragment ion mass tolerance of 0.60 Da and a parent ion tolerance of 10.0 parts per million. Carbamidomethylation of cysteine was specified in Mascot as a fixed modification. Gln->pyro-Glu of the N-terminus, deamidation of asparagine and glutamine, oxidation of methionine, dioxidation of methionine, and acetylation of the N-terminus were specified in Mascot as variable modifications.

### Criteria for Protein Identification and Quantitation

Scaffold (version Scaffold_4.4.5, Proteome Software Inc., Portland, OR) was used to validate MS/MS–based peptide and protein identifications. Peptide identifications were accepted if they could be established at greater than 95.0% probability by the Scaffold Local false discovery rate algorithm. Protein identifications were accepted if they could be established at greater than 99.0% probability and contained at least 2 identified peptides. Protein probabilities were assigned by the ProteinProphet algorithm (35). Proteins that contained similar peptides and could not be differentiated based on MS/MS analysis alone were grouped to satisfy the principles of parsimony. Proteins sharing significant peptide evidence were grouped into clusters. In addition, a label-free quantitative analysis was performed in Scaffold for those proteins (NEJ vs adult), with at least two unique peptides, that were present in all three biological replicates. The exponentially modified protein abundance index (emPAI) was used as a quantitative method with a t-test (Benjamini-Hochberg false discovery rate correction; significance level, p < 0.05) as a statistical method. For quantitation, missing values were replaced with a minimum value (default of zero), and normalization was performed with zero as a minimum value.

### Coproantigen ELISA

The coproantigen ELISA was tested against fecal samples taken from cattle naturally infected with *C. daubneyi* and with fecal egg counts ranging from 12 to 299 eggs per gram. Adult E/S products were used as a positive control. Fecal supernatants were prepared using a method adapted from Teimoori *et al* (36). Briefly, 1-g feces was homogenized in 3-ml lysis buffer (20-mM Tris HCl, 0.5% SDS, 8 M urea) and rotated overnight at RT (18–21 °C). The extracts were centrifuged at 8000*g* for 10 min, with the supernatant further centrifuged at 10,000*g* for 5 min and the final supernatant stored at −20 °C until use. Affinity-purified antibodies (anti–C. daubneyi E/S protein antibody [α-CdE/S]) were raised in a rabbit against whole E/S proteins collected from adult *C. daubneyi* maintained *in vitro* (Eurogentec). A proportion of these antibodies were biotinylated (biotinylated anti–*C. daubneyi* E/S protein antibody). The 96-well flat-bottom microtiter plates (Immulon 2HB, Thermo Scientific) were incubated with 50-μl (2 μg/ml) α-CdE/S Ig diluted in PBS per well and incubated overnight at 4 °C. Plates were washed six times in PBS containing 0.5% Tween 20 and then blocked for 1 h at RT in 1% skimmed milk/PBS-Tween. Fifty μl of the fecal supernatants was then added, at a dilution of 1:27 in PBS, and incubated for 2 h at RT. Plates were washed as before, and 50-μl (2 μg/ml) biotinylated anti–*C. daubneyi* E/S protein antibody added per well and incubated for 1 h at RT. Plates were washed as before, and 50 μl of ExtrAvidin–alkaline phosphatase conjugate (Sigma) (1:1500 dilution) was added per well. Plates were washed as before prior to the addition of 100 μl per well of SIGMAFAST p-nitrophenyl phosphate substrate (Sigma-Aldrich). After incubation at RT for 30 min, plates were read at 405 nm using a POLARstar Omega microplate reader (BMG LABTECH).

For assessment of specificity, 96-well plates were coated with 2 μg/ml of whole-worm extracts or E/S products (50 μl/well) from *F. hepatica* and a range of gastrointestinal nematode parasites that are commonly found in ruminants in the United Kingdom/Ireland (*Teladorsagia circumcincta*, *Ostertagia ostertagi*, *Haemonchus contortus*, *Trichostrongylus axei,* and *Trichostrongylus vitrinus*) and incubated overnight at RT. Plates were washed and blocked as described above, and then 2 μg/ml of α-CdE/S (50 μl/well) was applied for 2 h at RT. Plates were washed as before, and 50 μl per well of anti-rabbit immunoglobulin G/alkaline phosphatase conjugate (Sigma) was applied at a 1:1500 dilution. Plates were washed, developed, and read as described above. For all ELISAs, samples were analyzed in triplicate, and results are the representative of at least three independent experiments. ELISA results were considered positive for rumen fluke infection if returning an optical density greater than the mean plus two SDs of uninfected control samples. This cutoff was also used to define no cross-reactivity with the other species tested.

### Immunofluorescence Microscopy and Whole-Mount Preparations

Adult *C. daubneyi* and *F. hepatica* were fixed with 4% paraformaldehyde in PBS (Sigma-Aldrich) overnight at 4 °C and subsequently embedded in JB-4 resin (Sigma-Aldrich). Semithin sections, 2-μm thick, were cut on a pyramitome and mounted on clean glass slides. For immunofluorescence, JB-4 sections were washed with PBS and then incubated in 10 μg/ml of affinity-purified polyclonal α-CdE/S in an antibody diluent (AbD: PBS containing 0.2% (v/v) Triton X-100) overnight at 4 °C. As a negative control, comparable sections were incubated in rabbit preimmune serum. The sections were then washed three times in AbD before incubation in a 1:100 dilution of the secondary antibody, FITC-conjugated goat anti-rabbit immunoglobulin G (Sigma-Aldrich), in AbD for 1 h at RT. After three washes in PBS, the sections were mounted in glycerol containing 10% (v/v) PBS and 0.1 M propyl gallate (Sigma-Aldrich) and then viewed under a Leica DM2500 fluorescent microscope.

For whole-mount preparations, flukes were flat-fixed in formol acetic alcohol solution for 3 to 5 days at RT and then incubated in 1% (w/v) carmine (Sigma) for 1.5 h at RT. Flukes were then destained in 70% acidified ethanol overnight and dehydrated in a series of alcohols. The samples were then incubated overnight with HistoChoice clearing agent (Sigma) and mounted on glass slides using DPX mounting media (Sigma). Flukes were viewed using a Leica DM2500 microscope.

## Results

### The Intramammalian Life-Cycle Stages of *C. daubneyi*

**Fig. 1:**
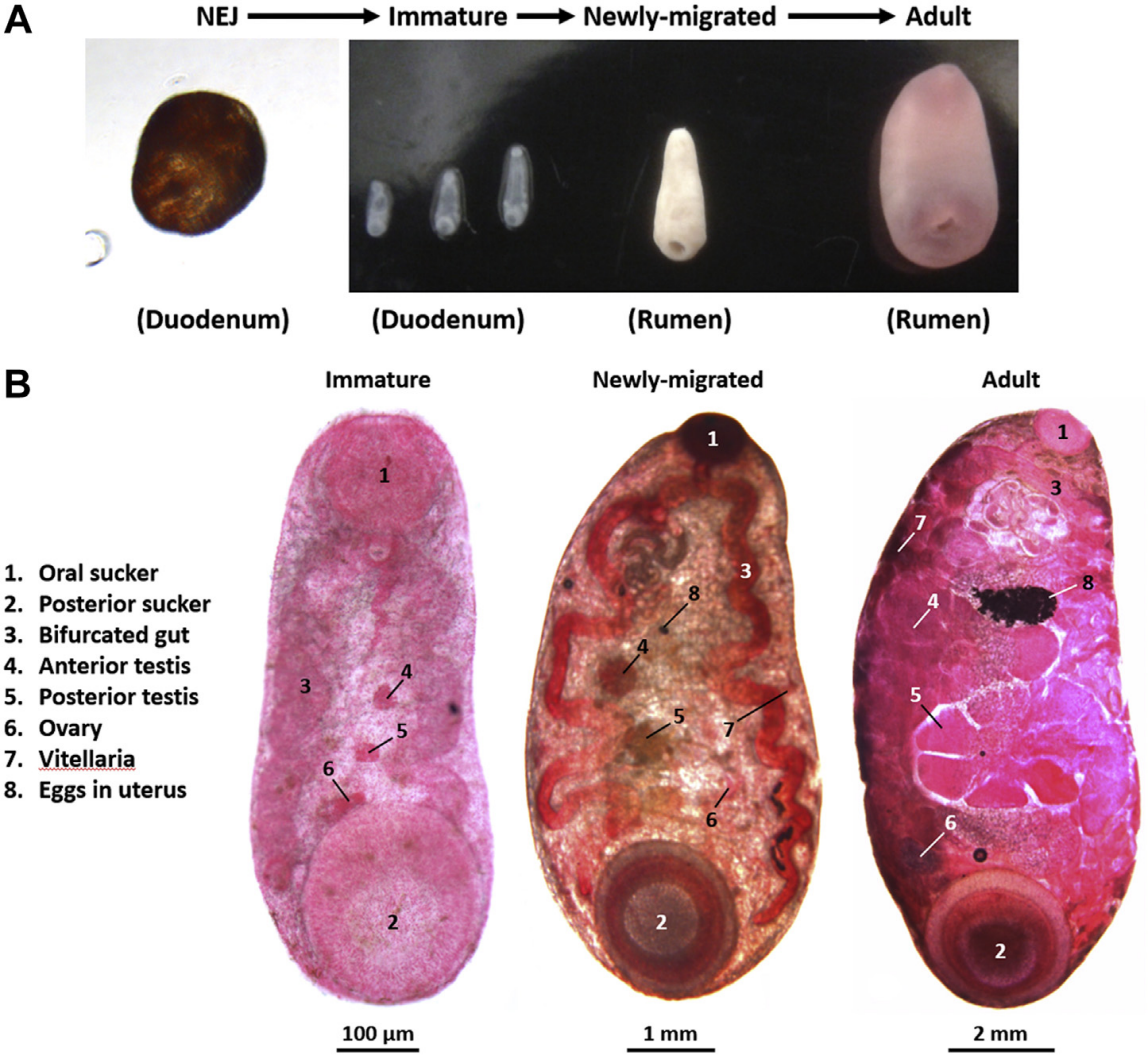
**The intramammalian life-cycle stages of *Calicophoron daubneyi*.***A*, four developmental stages of *C. daubneyi* were subjected to transcriptome analysis. These were as follows: newly excysted juveniles (NEJs) excysted here *in vitro* but typically found in the duodenum *in vivo*, immature flukes found embedded in the duodenal mucosa, small newly migrated flukes just arrived in the rumen, and fully developed adult flukes, also found in the rumen. *B*, carmine-stained whole immature, newly migrated, and adult flukes showing the major morphological features and internal organs.

The intramammalian life cycle of *C. daubneyi* involves four major developmental stages: (1) the NEJ flukes that emerge from ingested metacercariae and migrate into the duodenal submucosa, (2) the immature worms that actively feed on host tissue in the intestine, (3) small newly migrated flukes that have recently completed their migration to the rumen from the duodenum, and (4) mature adult flukes that are well established in the rumen. Macroscopic/microscopic examination of these stages shows their developmental progression (Fig. 1). The NEJs are typically 150 μm in length and already bear the oral and posterior suckers characteristic of paramphistomes. Histological examination revealed that while no reproductive structures were present, subtegumental muscle bundles and a developing bifurcated gut were observed (data not shown). The immature flukes (2–3 mm in length) are generally found in association with the duodenal mucosa though some remain within the intestinal lumen in very heavy infections. A prominent bifurcated gut was clearly visible in carmine-stained whole-mount worms as were genital anlagen corresponding to the rudimentary testes (anterior and posterior) and ovary. No vitellaria was observed at this stage (Fig. 1).

The newly migrated fluke (typically 5–7 mm in length) are found in the rumen attached to the rumen papillae, wall, or contents. Live worms are bright red in appearance and share similar morphology to adult fluke but are much smaller in size. In whole-mount preparations, the testes and ovary appear larger and more developed, and vitelline follicles have now appeared around the periphery of the fluke body. Despite their small size, eggs were often observed in the uterus of newly migrated flukes (Fig. 1). The eggs produced by newly migrated *C. daubneyi* were viable and released highly motile miracidia during *in vitro* egg hatch assays performed as described previously (37). Adult *C. daubneyi* (typically 1.5–2.0 cm in length) appear mid to pale pink with redder coloration around both the anterior and posterior suckers. They are found attached to the rumen wall or papillae *via* their posterior sucker. Whole-mount adult flukes showed fully developed reproductive structures with the posterior testis and egg-filled uterus being the most prominent features (Fig. 1).

### The *C. daubneyi* Transcriptome

**Fig. 2:**
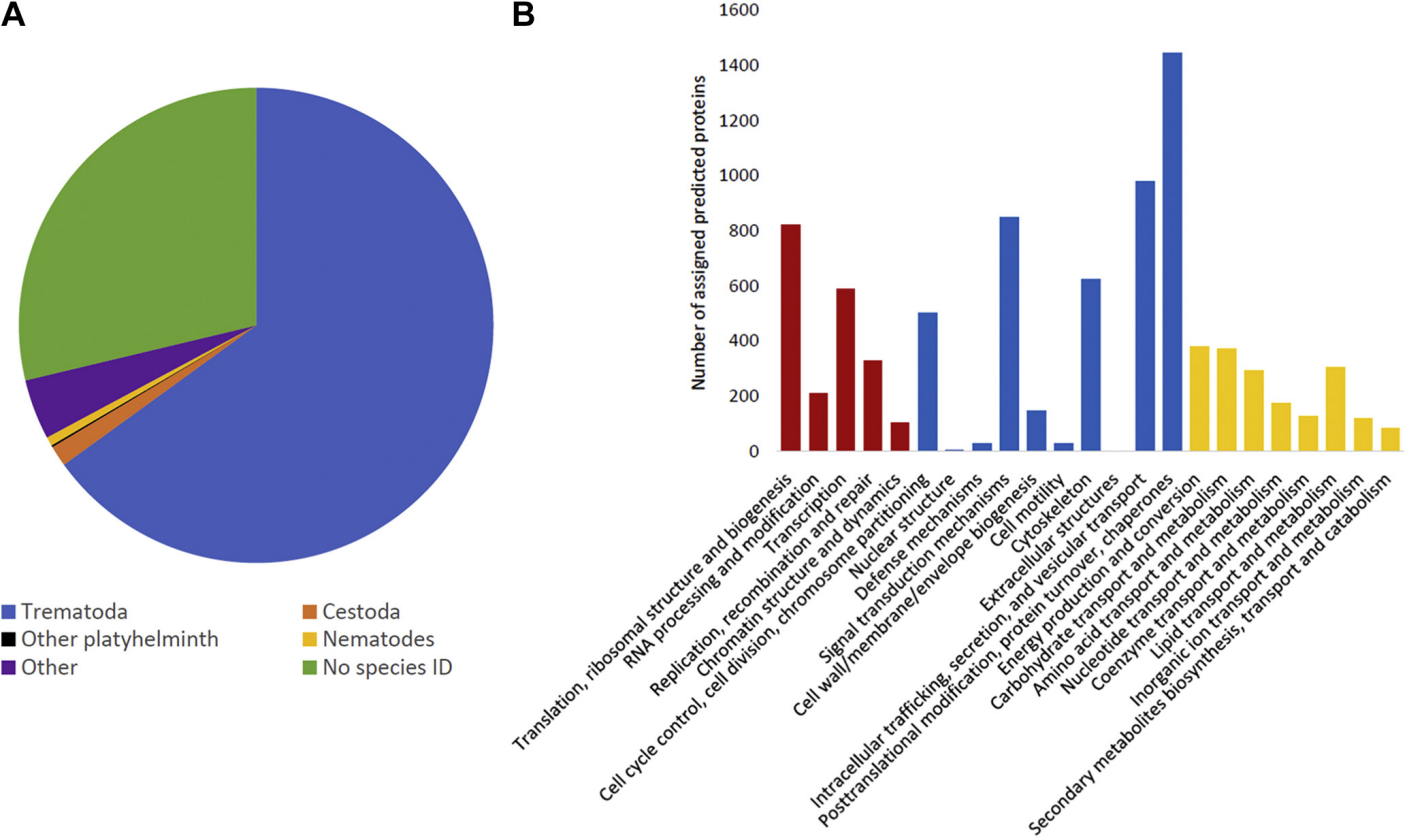
**Annotation of the *Calicophoron daubneyi* transcriptome.***A*, distribution of transcript annotation hits according to species. Analysis was performed using the top assigned annotation matches from the TREMBL Blast X annotation of all transcript sequences with a top-strand predicted protein sequence (24,956). *B*, eggNOG functional category annotations of predicted protein sequences returning an NOG/COG identification *via* the Trinotate annotation process (11,843/48,899 sequences). Categories are broadly grouped into genetic processes (*red bars*), cell structure and function (*blue bars*), and metabolism (*yellow bars*). The largest number of annotations was to the category ‘function unknown’ (4765 sequences), which are not shown.

The PacBio Iso-Seq approach generated a final dataset of 27,524 high-quality transcript sequences. Summary statistics for the Iso-Seq transcriptome sequencing and functional annotation are shown in Table 1. Functional annotation of the final 27,524 transcripts was performed using the Trinotate pipeline (20). A total of 17,793 transcripts (68.55%) with a top-strand protein prediction returned an assigned annotation using BlastX to interrogate the TrEMBL database. The majority of annotations returned were from related trematodes, with some hits from cestode and nematode species also. Only 4.1% of sequences returned an annotation to organisms outside of these taxonomic groups, while 7168 sequences could not be annotated. Annotated predicted top-strand protein sequences were grouped according to their eggNOG functional class, with intracellular trafficking/secretion/vesicle transport and post-translational modifications/protein turnover/chaperone classifications being particularly abundant (Fig. 2).

**Table 1:** Summary statistics for whole-transcriptome sequencing and assembly (PacBio Iso-Seq) and Trinotate functional annotation of transcript sequences for *Calicophoron daubneyi*

PacBio sequel library and Trinotate annotation	
Total transcripts after downstream processing (quality control) and cluster analysis	27,524
Total assembled bases (bp)	26,451,949
Average sequence length (bp)	961
Maximum sequence length (bp)	3452
Minimum sequence length (bp)	114
N50 (bp)	1128
GC content (%)	45.0
Transcripts with top strand protein prediction	24,956
Number of annotated top strand transcripts (Trinotate TREMBL BlastX) (%)	17,793 (68.55)
Proteins predicted (>50 AA)	48,899
Protein sequences with predicted signal peptide (%)	3696 (7.56)
Protein sequences with predicted transmembrane domain (%)	8373 (17.12)

### Expression Patterns of Transcript Functional Groups Are Associated With Parasite Development

**Fig. 3:**
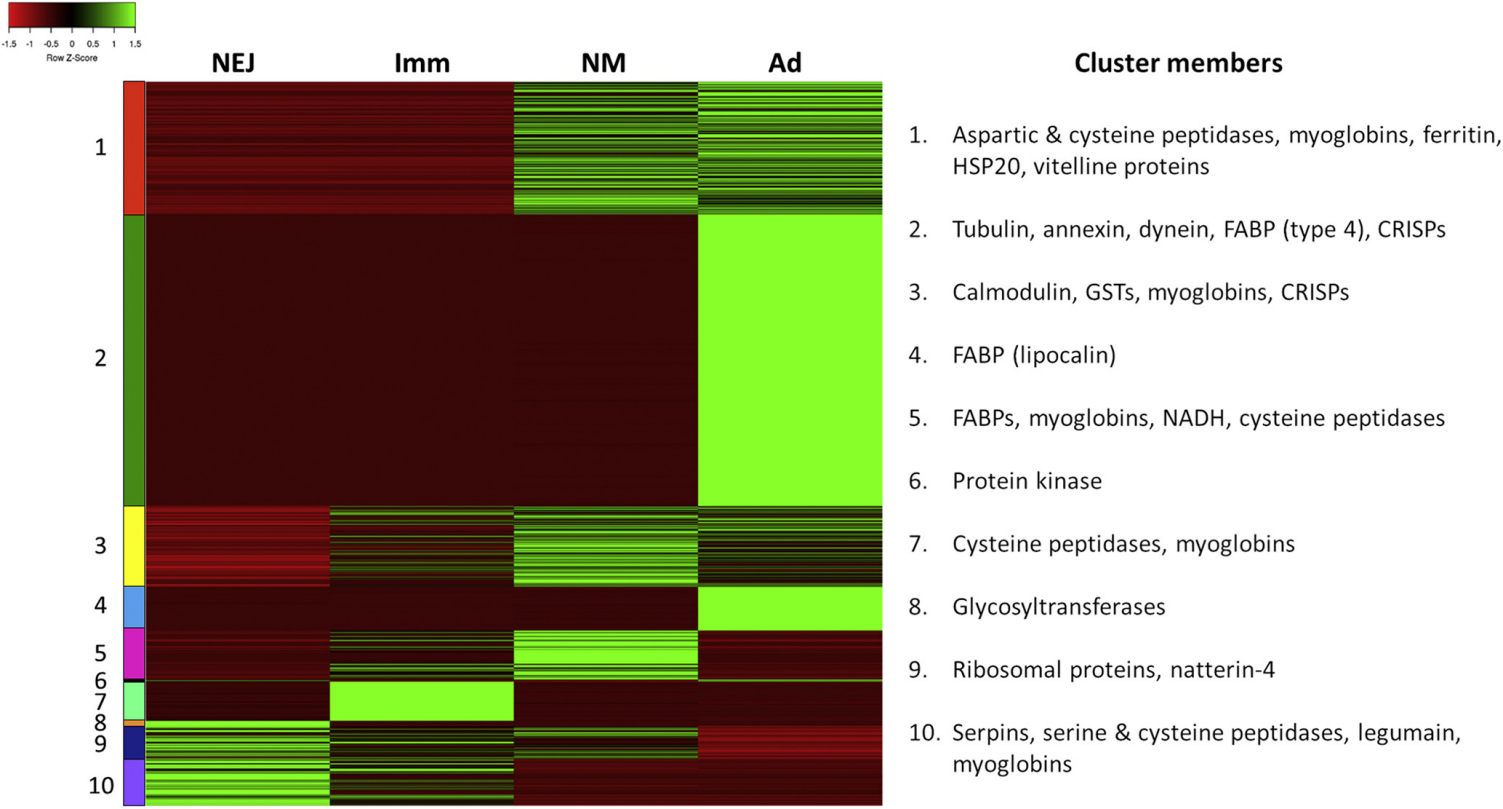
**Differential expression of *Calicophoron daubneyi* transcripts.** The top 10% of transcripts showing the most differential expression across our sample set were selected using a cut-off of at least a 9-log fold change in at least one comparison between *C. daubneyi* developmental stages and a minimum average TPM value of 2 in at least one stage. This returned 2470 transcripts (supplementary Table S1), which were grouped into clusters based on their expression pattern. A heat map of TPM values scaled by transcript values (where *red* indicates a low value and *green* a high value) was produced. Individual transcripts were matched to their BlastX annotation from the Trinotate output, and common proteins/protein family annotations present in each cluster (1–10) are highlighted. TPM, transcripts per million.

Mapping of the RNA-Seq Illumina reads to the full-length (Iso-seq) transcriptome-facilitated DE analysis across the four intramammalian life-cycle stages. Principle component analysis showed that there was clustering of the biological replicates but clear separation between each developmental stage (supplementary Fig. S1). An overview of the number of differentially expressed transcripts identified between each life-cycle stage is shown in supplementary Table S2. To identify transcripts with the greatest levels of DE across the four life-cycle stages, those with a logFC value of at least ± 9 in at least one pair-wise comparison across the whole dataset were selected. These sequences could be assigned to 10 distinct clusters based on their expression patterns (Fig. 3). The annotation for the top 10% DE transcript sequences is shown in supplementary Table S1. Notably, cysteine peptidases appeared in 4 of the 10 clusters, with each cluster having a different expression pattern, suggesting developmental regulation of individual enzymes (see below). Aspartic and cysteine peptidases as well as vitelline proteins were present in cluster 1, which is comprised of transcripts that are upregulated in the egg-producing newly migrated and adult stages. Transcripts encoding structural/cytoskeletal proteins including annexin, dynein, and tubulin were present in cluster 2, upregulated in the adult flukes. Transcripts encoding fatty acid–binding proteins (FaBPs) were assigned to clusters 2, 4, and 5 and showed upregulation in the newly migrated and adult stages. Oxygen-scavenging myoglobins were represented in clusters 3, 7, and 10. In the latter group, transcripts encoding serine proteases and serine protease inhibitors were prevalent, indicating their upregulation in the NEJ and immature stages of *C. daubneyi*. Cysteine-rich secretory proteins were also prominent in clusters 2 and 3 with upregulation in adult and newly migrated stages, respectively.

Major metabolic pathways used by parasitic helminths also showed considerable changes in expression across the intramammalian stages of *C. daubneyi.* Initial BLAST analysis revealed that transcripts encoding key enzymes of the glycolysis/gluconeogenesis, tricarboxylic acid (TCA) cycle, and malate dismutation pathways were conserved in *C. daubneyi.* We found that most members of the glycolysis/gluconeogenesis pathway showed the highest expression in the adult flukes. In contrast, the majority of TCA cycle members were expressed more highly by NEJ/early stages. Finally, expression of transcripts encoding members of the malate dismutation pathway gradually increased with fluke development and showed significant upregulation within the adult stage (supplementary Fig. S2).

### *C. daubneyi* Expresses Orthologues of Helminth Immunomodulators During Acute and Chronic Infection

A defining feature of helminths is their ability to modulate the host immune response to promote their longevity and reproduction. While many individual molecules with reported immunomodulatory activity have been identified from a range of trematode species, we know very little about the secretion of such molecules by paramphistomes. To begin to address this, we used the sequences of known trematode immunomodulators as queries for BLASTP analysis to determine whether orthologues were expressed by *C. daubneyi*. BLAST searches using the trematode-specific helminth defense molecules (HDMs; (38)) as queries identified 13 transcripts. Phylogenetic analysis showed that these sequences separated into 3 distinct clades, each containing/encoding one dominant isoform (based on TPM values). Members of these clades were termed CdHDM-1, CdHDM-2, and CdHDM-3, respectively (supplementary Fig. S3). To determine the expression pattern for the HDM orthologues across the *C. daubneyi* life stages, the sum of the TPM values for members of each clade (1, 2 and 3) was calculated. This revealed that HDM transcripts were more highly expressed in the NEJ and immature stages than in the rumen-dwelling newly migrated and adult stages with the exception of clade 2 CdHDMs, which were also upregulated in the newly migrated stage.

Proteins encoded by members of the CdHDM transcript clades were also identified in the secretomes of NEJs and adult flukes by MS. Although CdHDM-2 and CdHDM-3 showed lowest transcription in the adult flukes compared with the other life-cycle stages, a CdHDM-3 clade member was found to be the most abundant protein detected in adult *C. daubneyi* E/S when analyzed by LC-MS/MS. Indeed, both CdHDM-2 and CdHDM-3 proteins were detected in adult and NEJ E/S preparations. Helical wheel analysis indicated that only CdHDM-2 and CdHDM-3 contained the conserved C-terminal amphipathic helix, which is a key functional feature of this group of immunomodulators (38, 39, 40) (supplementary Fig. S3).

Orthologues of other trematode immunomodulators, including kunitz-type inhibitor, peroxiredoxin, FaBPs, venom allergen–like 9/13, and glutathione-*S*-transferase (GST) Sigma class, were also identified after BLAST analysis. At the transcript level, most of these molecules showed the highest expression in *C. daubneyi* NEJs while some (*e.g.*, GST sigma and FABP 2/15) showed the highest expression in the immature and/or newly migrated stage (Fig. 3).

### Qualitative and Quantitative Analysis of the Secretome of *C. daubneyi* NEJs and Adult Flukes

E/S proteins released by *C. daubneyi* NEJs and adult flukes during *in vitro* cultivation were concentrated from culture supernatants and analyzed by LC-MS/MS against our *C. daubneyi* Iso-Seq transcriptome. For this analysis, a protein match was only accepted if all 3 biological replicates contained 2 or more unique matched peptides. A total of 506 proteins were identified in the NEJ E/S and 540 proteins in the adult E/S after applying these stringent criteria (supplementary Table S3). Of these, 272 proteins were unique to the NEJ secretions, 306 unique to the adult secretions, and 234 proteins identified in both samples.

**Fig. 4:**
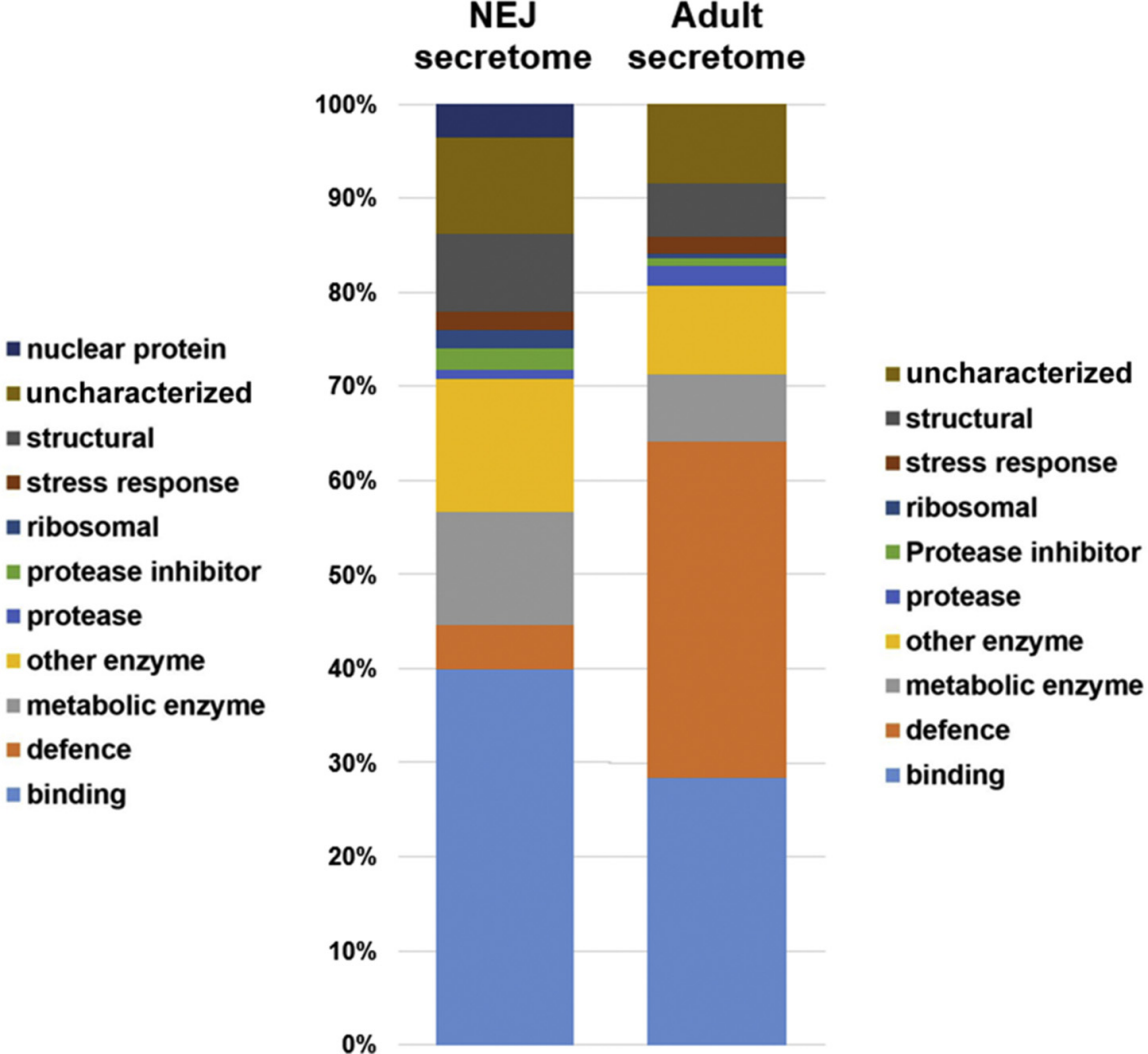
**Comparative analysis of the secretome of *Calicophoron daubneyi* NEJs and adult flukes.** Graphical representation of the composition of the top 50 most abundant secretory proteins (based on emPAI scores) grouped according to the function. emPAI, exponentially modified protein abundance index; NEJ, newly excysted juvenile.

The top 50 most abundant proteins (based on emPAI scores) in the NEJ and adult E/S samples were grouped into functional categories (Fig. 4). In the NEJ secretome, proteins with binding functions were particularly abundant, representing nearly 40% of the identified proteins. This group included several myoglobins, which act as oxygen scavengers, and a number of calcium-binding proteins. Approximately 5% have roles in defense, such as the GSTs and CdHDM-2. Metabolic/other enzymes and structural (cytoskeletal and membrane) proteins comprised a large proportion of the top 50 most abundant proteins, as did a number of uncharacterized proteins.

In the adult secretome, defense-associated proteins were especially abundant, representing over 35% of the total proteins in this group. As stated above, a CdHDM-3 clade member was the single most abundant protein based on the emPAI value. Other molecules categorized as ‘defense’ belonged to the GST family. A smaller proportion of the top 50 most abundant adult E/S proteins have roles in binding, and other functional groups are of similar proportions to those seen in the NEJ secretions.

**Fig. 5:**
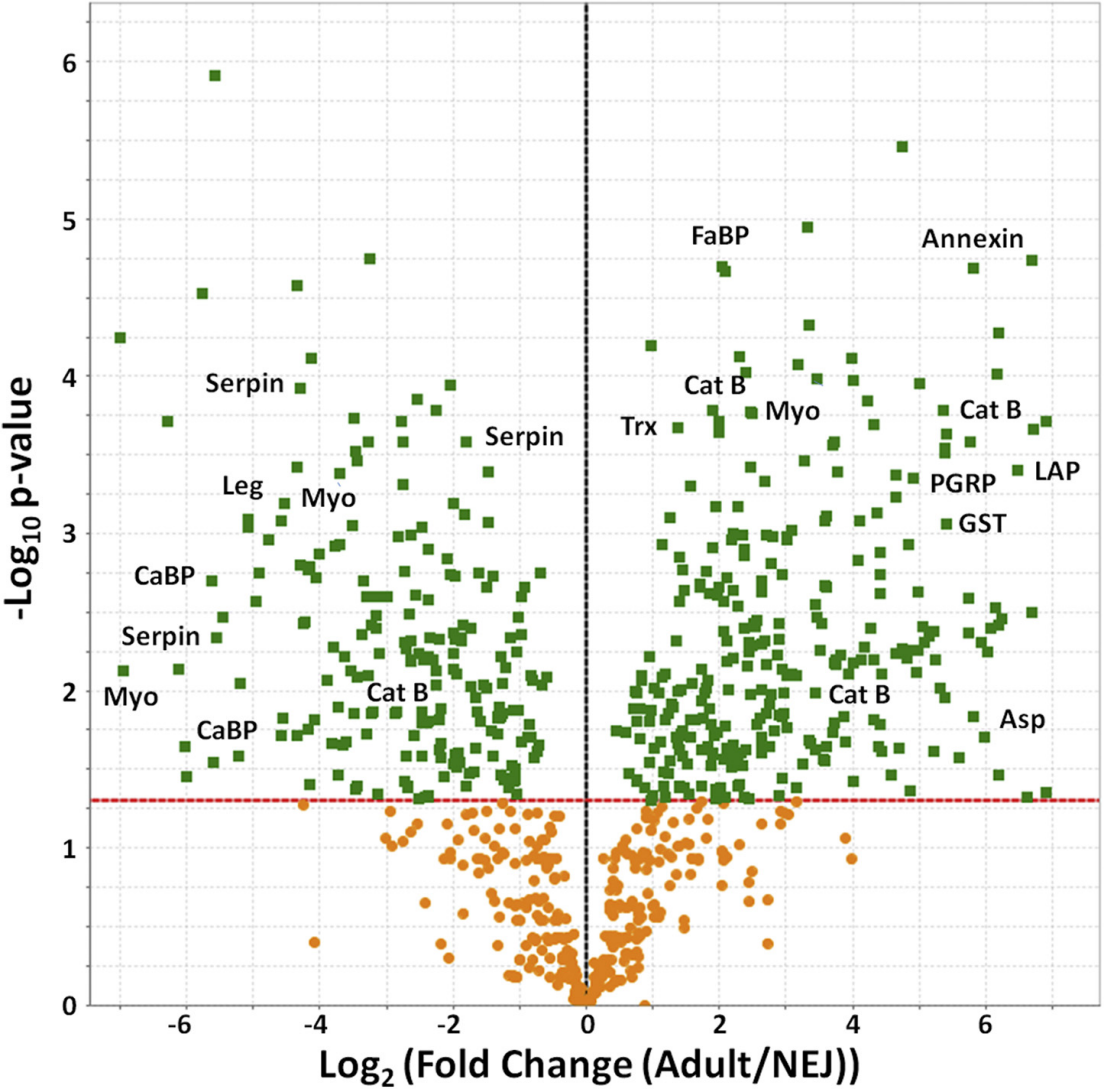
**Qualitative and quantitative proteomics analysis reveals developmental expression of proteins secreted by NEJ and adult *Calicophoron daubneyi*.** Proteins identified in the secretome of *C. daubneyi* NEJs and adult flukes (*>*2 matched peptides in 3 out of 3 biological replicates) were subjected to quantitative analysis shown as a Volcano plot. The x-axis represents log_2_ (fold-change) (adult/NEJ), and the y-axis shows the −log_10_ (*p* value). The *dashed red line* indicates the significance threshold (*p* = 0.05). The *vertical dashed black line* indicates zero fold change. *Green boxes* represent proteins whose expression is significantly different between the two life stages. The position of selected proteins is shown. Asp, aspartic protease; CaBP, calcium-binding protein; Cat B, cathepsin B; FaBP, fatty acid–binding protein; LAP, leucine aminopeptidase; Leg, legumain; Myo, myoferlin; NEJ, newly excysted juvenile; PGRP, peptidoglycan-recognition protein; Trx, thioredoxin.

A label-free quantitative approach (based on emPAI values) was used to determine the respective levels of the proteins secreted by *C. daubneyi* NEJs and adult flukes. The volcano plot representation of the data identified several proteins such as serpins, myoglobins, and legumain that were significantly (*p* < 0.05) enriched in the NEJ secretome (Fig. 5). These molecules were also members of the NEJ-associated transcript group 10 (Fig. 3), confirming their expression/secretion by NEJs at the protein level. In contrast, aspartic proteases, cathepsin B peptidases, and myoglobin (transcript group 1; Fig. 3) and annexins and FaBPs (transcript group 2; Fig. 3) were significantly enriched (*p* < 0.05) in the adult secretome (Fig. 5). Other proteins enriched in the adult fluke secretome, with potential roles in defense, included GST, thioredoxin, and peptidoglycan-recognition protein. These results show that *C. daubneyi* secretory proteins are developmentally regulated and correlate with the migration of the fluke within the gastrointestinal tract of the ruminant host.

The top 50 most abundant proteins in the NEJ and adult *C. daubneyi* secretomes (as described above) were compared with the top 50 proteins secreted by *F. hepatica* NEJs and adult flukes. *F. hepatica* was chosen for comparative analysis as it is frequently found coinfecting the same ruminant host as *C. daubneyi* and because the secretions from both species were collected at the same time points after *in vitro* culture, 24 h for NEJs (41) and 5 h for adult flukes (42). At the NEJ stage, there was a 24% (12/50) overlap with actin, cathepsin B, enolase, fasciclin-1, FaBPs, fructose-bisphosphate aldolase, GST, myoglobin, nucleoside diphosphate kinase, protein disulfide isomerase, serpin, and superoxide dismutase present in both species. For the adult stages, there was a 20% (10/50) overlap with annexin, cathepsin B, enolase, FaBPs, fructose-bisphosphate aldolase, GST, HDM, legumain, myoglobin, and triosephosphate isomerase detected in the secretome of both species.

### Cysteine Peptidase Families Have Undergone Selective Expansion in *C. daubneyi*

Cathepsin-like cysteine peptidases dominate the transcriptome and secretome of many trematode species (43, 44). Accordingly, we used the primary amino acid sequences of cathepsins B, D, F, and L from *Schistosoma mansoni*, *O. viverrini*, *F. hepatica,* and *C. sinensis* as queries for BLAST analysis of our *C. daubneyi* transcriptome. Using this approach, a single transcript (Cdaub_01,253) encoding a 53.0-kDa cathepsin F–like cysteine peptidase was identified. InterPro analysis showed that the molecule possessed the N-terminal proteinase inhibitor (prosegment) domain (IPR013201) and the C-terminal C1A peptidase domain (IPR000668), which are typical of cysteine peptidases. An additional cystatin domain (SSF5440) was present N-terminal to the prosegment and contained a partial QXVXG motif (QAPGG) required for inhibition of cysteine peptidases (45). In the prosegment region, the ERFNAQ-like and E/DXGTA motifs characteristic of cathepsin Fs (46, 47) were present, while the GXNXFXD-like motif required for pH-dependent autoprocessing of cysteine peptidases (48) was partially conserved (GITPFSD).

A further two transcripts (Cdaub_01489 and Cdaub_08378) encoding cathepsin L-like cysteine peptidases were also identified although the latter was truncated at the 5` end. For the full-length sequence, InterPro analysis confirmed the presence of the C-terminal peptidase domain (IPR000668) and the N-terminal prosegment (IPR013201). Although the GXNXFXD-like motif required for pH-dependent processing was fully conserved (GVNEFSD), conserved asparagine residues found at the boundary of the prosegment/mature peptidase domain (allowing initial *trans-*activation by asparaginyl endopeptidase/legumain) in other trematode cathepsin Ls (49) were absent. Furthermore, a specific LSH cleavage site required (in *F. hepatica*) for subsequent removal of the prosegment of cathepsin L zymogens by *trans-*activated cathepsin L molecules (50) was not conserved in the *C. daubneyi* cathepsin L sequence. The S2 subsite, a binding pocket that governs substrate specificity of cysteine peptidases, is composed of residues occupying positions 67, 68, 133, 157, 160, and 205 with positions 67, 157, and 205 being particularly important (51). The key S2 subsite residues were found to differ between the 2 *C. daubneyi* cathepsin L–like peptidases: Cdaub_01489 (S2 sub-site: Leu^67^, Leu^157^ and Leu^205^) and Cdaub_08,378 (S2 sub-site: Tyr^67^, Met^157^ and Ala^205^) indicating that they will have distinct substrate preference.

**Fig. 6:**
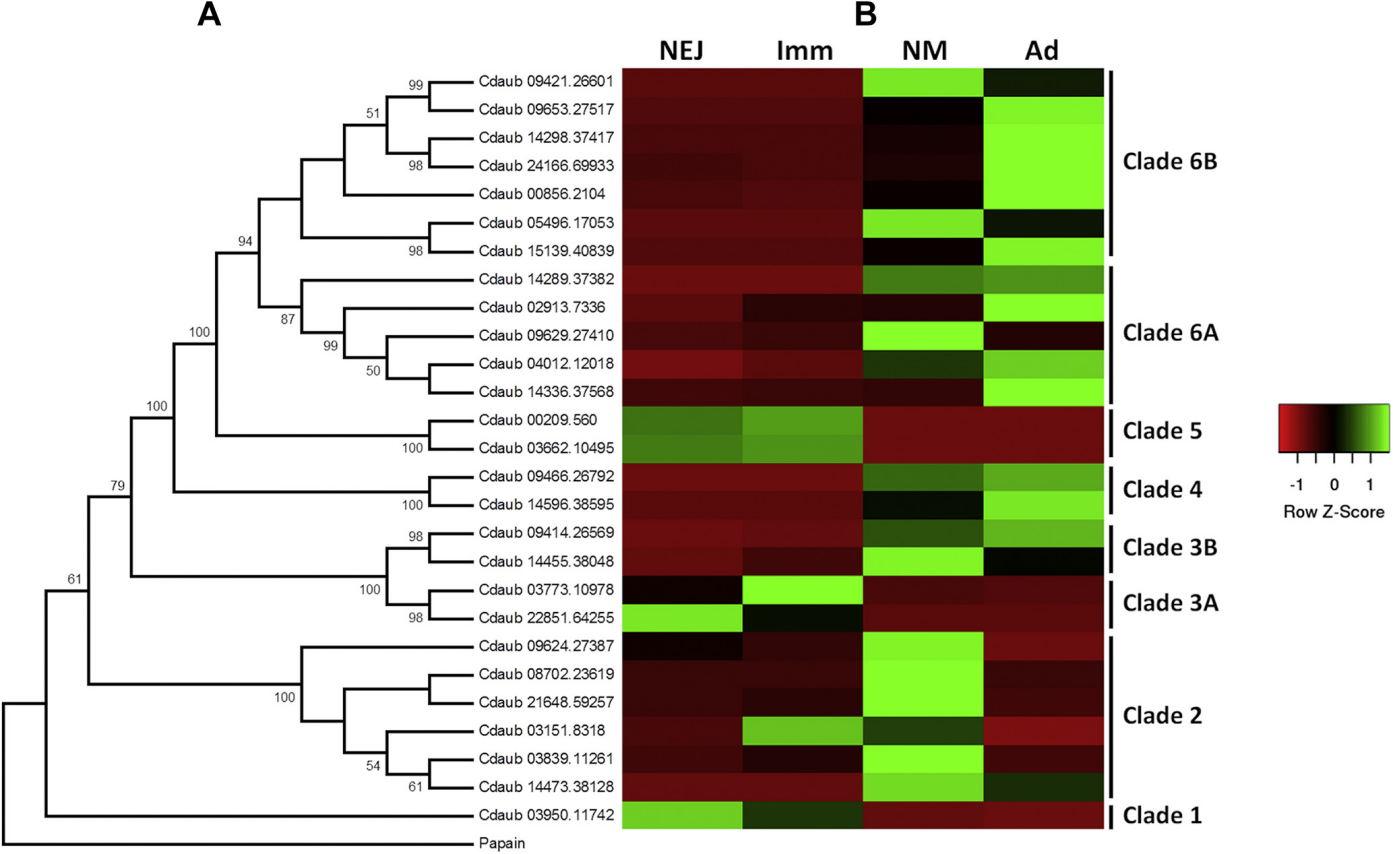
**Phylogenetic relationships of the *Calicophoron daubneyi* cathepsin B family and their developmental expression patterns.***A*, bootstrapped (1000 trials) neighbor-joining phylogenetic tree showing the evolutionary relationship of *C. daubneyi* cathepsin B amino acid sequences. Numbers represent bootstrap values (given as percentages) for a particular node and values greater than 50% are shown. The tree is rooted to *Carica papaya* papain (UniProt accession P00784). *B*, the TPM values of transcripts encoding the cathepsin B clade members were represented as a heat map to visualize their expression patterns across the intramammalian life-cycle stages of *C. daubneyi*. Relative expression is shown by a *red* to *green* scale depicting low to high levels of expression, respectively. TPM, transcripts per million.

In contrast to the cathepsins F and L, BLAST analysis found significant expansion of *C. daubneyi* transcripts encoding cathepsin B and aspartic proteases (cathepsin D). A total of 27 cathepsin B transcripts were identified. A phylogenetic analysis of the *C. daubneyi* cathepsin B protein sequences revealed that they separated into six well-supported clades (Fig. 6*A*). Mapping of TPM values against the transcripts showed that the clade members are developmentally expressed with clades 1, 3A, and 5 showing the highest expression in the intestinal NEJ and immature stages, while members of clades 2, 3B, 4, 6A, and 6B are expressed predominantly by rumen-dwelling newly migrated and adult flukes (Fig. 6*B*). As for cathepsin L cysteine peptidases, the substrate specificity of cathepsin Bs is also determined by the composition and arrangement of amino acids that create the S2 active site subsite. Using primary sequence alignments and analysis of the atomic structure of *S. mansoni* cathepsin B, the key residues in this pocket that interact with the P2 amino acid of the substrate have been identified as those situated at positions 100, 146, 244, 245, 269, and 316 (52). A comparison of the amino acids that occupy these positions in the various phylogenetic clades of the *C. daubneyi* cathepsin B family is shown in Table 2 and reveals a number of substitutions that could have a critical impact on their substrate preferences.

**Table 2:** Comparison of the residues from the S2 active site in *Calicophoron daubneyi* cathepsin Bs (clades 1–6)

Clade	Position					
	100	146	244	245	269	316
1	Cys	Pro	Thr	Phe	Ala	Ser
2	Cys	Pro	Asp	Phe	Gly	Asp
3A	Cys	Ser	Ala	Tyr	Gly	Asp
3B	Cys	Pro	Ala	Phe	Gly	Asp
4	Cys	Leu	Gly	Phe	Asn	Phe
5	Cys	Asp	Ser	Leu	Tyr	Arg
6A	Cys	Asp	Ser	Phe	Tyr	Asp
6B	Cys	Asp	Tyr	Phe	Tyr	His

Residues were identified using primary sequence alignments and analysis of the atomic structure of *Schistosoma mansoni* cathepsin B (52; Protein Data Bank codes 3QSD, 3S3Q, and 3S3R).

Similarly, a family of 28 aspartic proteases were identified after BLAST analysis of the *C. daubneyi* transcriptome. Phylogenetic analysis segregated the sequences into 7 clades (supplementary Fig. S4*A*), which again displayed developmental regulation: while the expression was low for all aspartic proteases in the NEJ stage, clade 3 enzymes and two members of clade 7 showed the highest expression in the immature flukes. The remaining clade members showed clear upregulation in the newly migrated and adult worms (supplementary Fig. S4*B*).

At the protein level, cysteine peptidases were identified in the secretions of *C. daubneyi* NEJs and adult flukes although, based on emPAI values, they did not appear to be the dominant molecules: only 2 of the 6 cathepsin Bs identified in the NEJ secretions were in the top 50 most abundant proteins. For the adult secretome, it was 5 of 15. Similarly, none of the 2 aspartic proteases identified in the NEJ secretions were in the top 50 proteins, and for the adult secretome, it was 1 of 11. The cathepsin F, and both cathepsin L peptidases, were identified in the secretome of *C. daubneyi* NEJs while a single cathepsin L (Cdaub_01489) was also present in adult fluke secretions (none were present in the list of top 50 proteins).

### Development of a Coproantigen-Based ELISA for Detection and Diagnosis of *C. daubneyi*

**Fig. 7:**
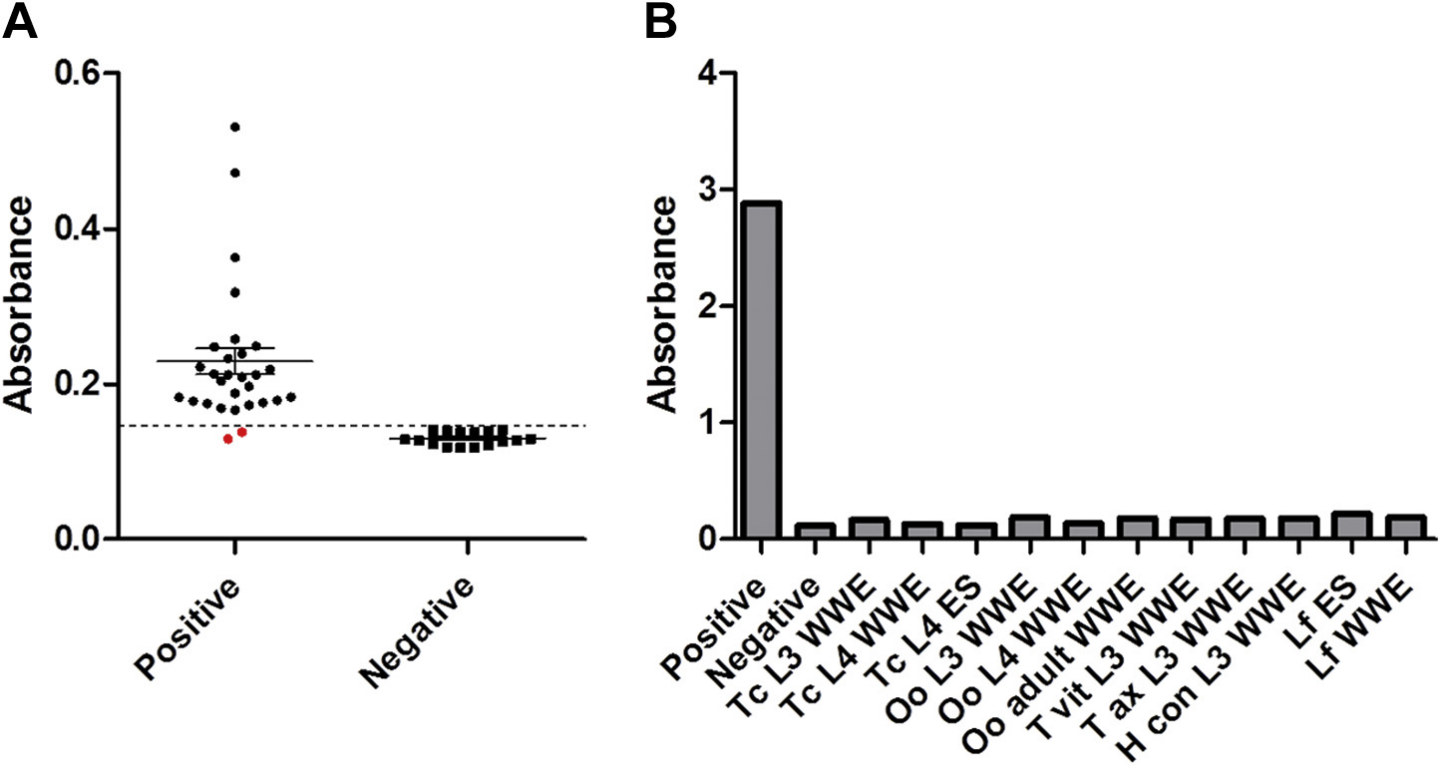
**Confirmation of *Calicophoron daubneyi* infection by coproantigen ELISA.***A*, fecal samples were prepared from cattle independently determined, by fecal egg count, to be infected or uninfected by *C. daubneyi*. A sandwich ELISA approach was used with native and biotinylated polyclonal antibodies raised against whole adult *C. daubneyi* E/S proteins. Absorbance at 405 nm of infected and uninfected samples. *p* value from *t*-test. The hatched line shows absorbance two SDs greater than mean uninfected absorbance; *red dots* indicate samples from infected animals below this limit. *B*, specificity of anti-*C. daubneyi* antibodies by ELISA against indicated parasites and life stages. E/S, excretory/secretory.

A coproantigen-based sandwich ELISA was developed to assess the potential of E/S for diagnosis of *C. daubneyi* infection in ruminant livestock. Anti–Cd-E/S antibodies raised in rabbits were coated on ELISA plates and used to capture *C. daubneyi* antigens present in fecal supernatants from naturally infected cattle. This approach gave high sensitivity of diagnosis (93%), with 28 of 30 samples from infected animals showing reactivity greater than 2 SDs of the mean uninfected absorbance (Fig. 7*A*). To determine the limit of detection of the assay, adult *C. daubneyi* E/S products were serially diluted (from 3 mg/ml) and tested in the antigen-capture ELISA. Taking the limit of detection to be two SDs above the mean background absorbance, regression against the linear portion of the dilution series yielded a limit of detection of 7.0 ± 0.17 ng/ml. The specificity of the assay was assessed by ELISA by incubating the α-Cd-E/S antibodies with E/S or whole-worm extracts from the liver fluke, *F. hepatica*, and a number of gastrointestinal nematode parasites commonly found in ruminants in the United Kingdom/Ireland (*T. circumcincta*, *O. ostertagi*, *H. contortus*, *T. axei,* and *T. vitrines*). No cross-reactivity was observed (Fig. 7*B*).

**Fig. 8:**
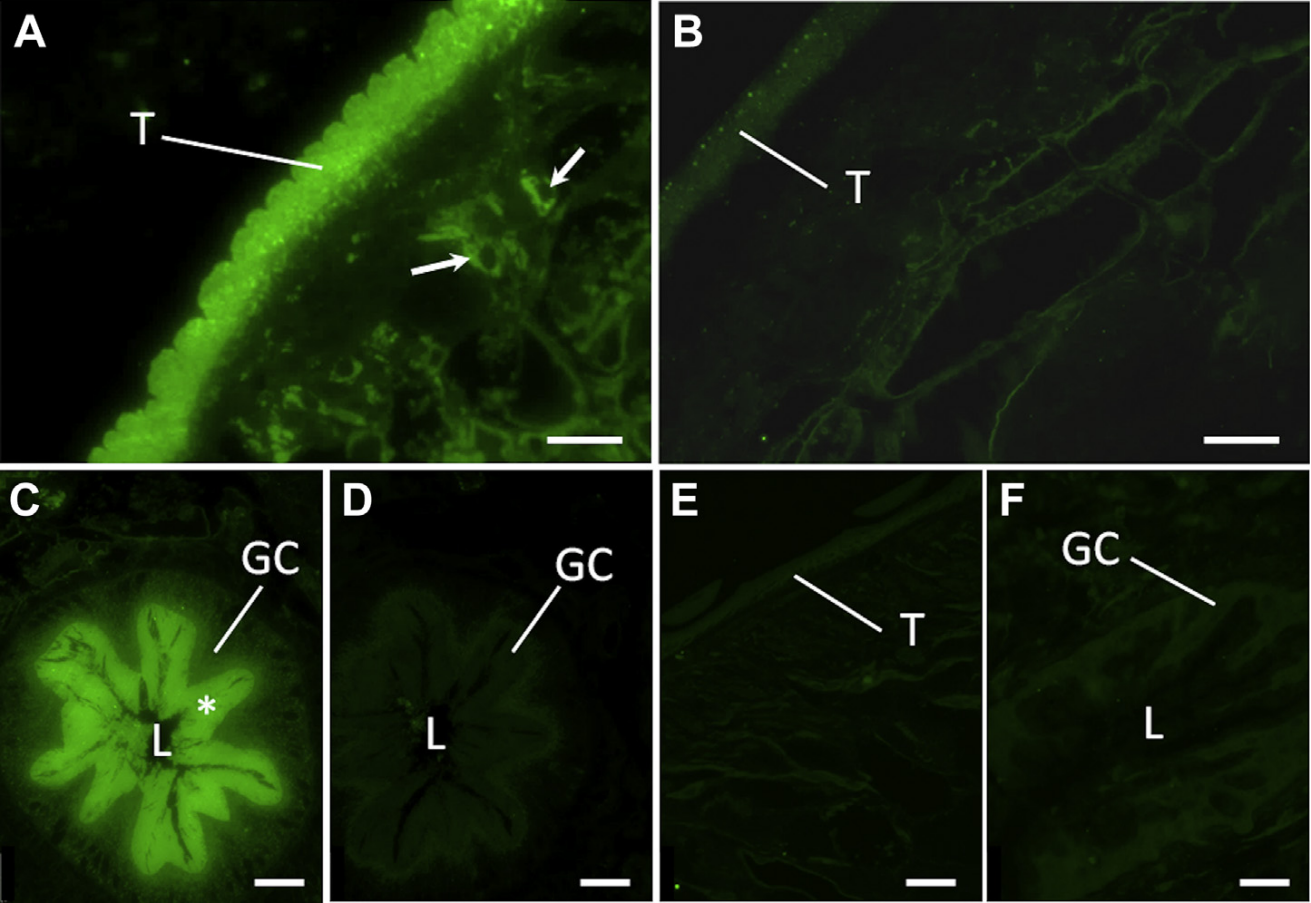
**Light micrographs showing α-CdE/S immunofluorescence in sections of adult *Calicophoron daubney*i.***A*, the tegumental syncytium (T) and underlying tegumental cell bodies (*arrows*) of *C. daubneyi* show intense and specific labeling with the α-CdE/S antibody. *B*, *C. daubneyi* control section which was incubated in preimmune rabbit serum. All tissues, including the tegument, are unlabeled. *C*, section showing specific labeling of the lamellae (∗) that arise from the gastrodermal cells (GCs) that line the fluke gut. *D*, *C. daubneyi* control section which was incubated in preimmune rabbit serum shows no immunofluorescence. *E* and *F*, sections of adult *F. hepatica* show no labeling of the tegument (T) or gastrodermal cells (GCs) when probed with the α-CdE/S antibody. *Scale bars* represent 25 μm. L, gut lumen; α-CdE/S, anti–*C. daubneyi* excretory-secretory protein antibody.

Immunofluorescence was used to determine the origin of the secreted antigens recognized by the α-Cd-E/S antibodies (Fig. 8). Sections of adult *C. daubneyi*, probed with the α-Cd-E/S antiserum, showed strong immunolabeling of the tegumental syncytium and underlying tegumental cell bodies. Intense immunoreactivity was also observed in the lamellae that project from the gastrodermal cells that line the fluke gut. In contrast, the α-Cd-E/S antiserum did not recognize equivalent tissues from *F. hepatica,* corroborating the ELISA results. No immunolabeling was observed when sections were probed with preimmune sera.

## Discussion

Despite the rise in prevalence of *C. daubneyi* across much of Western Europe (1) and the sporadic outbreaks of acute, fatal, paramphistomosis (*e.g.*, (4)), very little is known about the basic biology and pathogenicity of this species. To begin to address this, we have produced the first transcriptome datasets for four intramammalian life-cycle stages of *C. daubneyi* and characterized the secretome of the NEJ and adult flukes that are responsible for acute and chronic diseases, respectively. In doing so, we have provided the first description of how various metabolic, virulence, and invasive factors are regulated with parasite development and have used these molecules to develop the first ELISA-based coproantigen test for diagnosis of *C. daubneyi* infection of livestock.

Transcriptome data are currently available for two paramphistome species, *Paramphistomum cervi* and *C. daubneyi* (53, 54). However, these studies have been limited to the adult life-cycle stage, which are often described as “well-tolerated” by their ruminant hosts because they are responsible for chronic paramphistomosis, which is largely subclinical (13). Here we present a comparative transcriptome analysis of the adult stage with the previously uncharacterized NEJs (infective stage), immature flukes, and newly migrated flukes (migratory stages). We initially used the PacBio platform to generate full-length transcripts for all four life-cycle stages without the need for troublesome assembly of short reads. PacBio sequencing identified a total of 24,956 full-length transcripts with a top-strand protein coding region. This method almost certainly avoids transcript overestimation, with the number of transcripts described here for *C. daubneyi* approximately half of those reported for other trematode parasites using *de novo* assembly (*e.g.* (53, 54, 55, 56)) but similar to those which have been produced using genome-guided approaches (57, 58, 59).

Changes in gene transcription were followed across the four intramammalian developmental stages of *C. daubneyi* using TPM values derived from RNA-Seq libraries (in biological triplicate for each stage). Principal component analysis revealed clustering of the replicates but clear separation between life-cycle stages. This demonstrates that each life-cycle stage displays distinct patterns of temporal gene expression that correlate with both their development/maturity and niche within the mammalian host. DE analysis revealed that the most highly regulated transcripts (*i.e.*, those showing at least a 9-log fold change in at least one comparison between stages) were dominated by a few functional groups. Some of the most striking transcriptional changes involved upregulation in both the newly migrated and adult stages. These included tubulins and dynein, both key structural components of the axoneme of trematode spermatozoa (60, 61), and the vitelline proteins that comprise trematode eggshells (62). This temporal expression pattern of reproduction-associated genes follows the appearance of the reproductive organs during *C. daubneyi* development with the anterior and posterior testes, ovary, and vitellaria first seen (all together) by the newly migrated stage. Despite the clear size difference between the newly migrated and adult rumen fluke, both stages produced eggs. Thus, it is likely that egg production begins rapidly upon the arrival of the migrating flukes into the rumen, similar to the almost immediate onset of egg production observed when juvenile *F. hepatica* enter the bile duct from the liver parenchyma (61).

A total of 7.5% of our predicted protein sequences possessed an N-terminal signal peptide for secretion *via* the endoplasmic reticulum/Golgi pathway, which is comparable with other trematodes such as *C. sinensis* (6.5%) and *O. viverrini* (6.9%) (56). Indeed, the majority of the most highly regulated transcripts encoded proteins found in *C. daubneyi* secretions after the LC-MS/MS analysis. Moreover, our quantitative proteomics analysis showed that many proteins including myoglobins, serpins, cysteine peptidases, aspartic peptidases, GSTs, and FaBPs were developmentally regulated, which corroborates the transcriptome analysis. The secretomes of two key stages in the *C. daubneyi* intramammalian life cycle were analyzed: the infectious NEJs that are responsible for acute disease in the duodenum and the mature adult worms found in the rumen during chronic infection. Proteins with binding function dominated the secretions of both stages, notably 14-3-3 proteins, calcium-binding proteins, FaBPs, ferritin, and myoglobin isoforms. Although some of these molecules have reported immunomodulatory functions in other trematodes (63, 64, 65), their capacity for binding would also allow them to function as carriers to facilitate uptake of various molecules from the host microenvironment *in vivo.* Interestingly, ferritin was enriched in adult fluke secretions compared with those of the NEJ. Ferritin is believed to act as an iron carrier after extracellular hemoglobin digestion in the schistosome gut (66). Because adult *C. daubneyi* are not thought to feed directly on host blood (because of the absence of supporting pathology) (1) secreted ferritin may sequester iron from rumen fluid or have alternative immunomodulatory roles as described for *C. sinensis* (67). Similarly, myoglobin was highly expressed by *C. daubneyi* at the transcript level, and different isoforms were found to be enriched in NEJ and adult fluke secretions. Myoglobins act as oxygen scavengers and are thought to be important for parasite metabolism through oxygen transport/storage (68, 69). Trematode myoglobins have some of the highest recorded affinities for oxygen (70), which would be of particular value in the largely anaerobic rumen (71).

Metacercariae are nonfeeding stages and must rely upon endogenous glycogen stores to support them during excystment and initial establishment of infection. In *F. hepatica,* the relative expression levels of key enzymes that regulate glycogen synthesis and degradation indicate that metacercarial glycogen stores are rapidly used up after excystment (41). Similarly, fructose-1,6-bisphosphatase (involved in glycogen synthesis) and phosphofructokinase (involved in glycogen degradation) were both upregulated in *C. daubneyi* NEJs, indicating that the fluke is expending pre-existing glycogen stores while simultaneously replenishing them during early infection. Conceivably, this provides the NEJ with sufficient energy to establish infection (*e.g.*, to support active secretory processes and locomotion required during penetration into the duodenal mucosa) while creating new reserves to metabolize during the long migration toward the rumen.

Trematodes undergo drastic changes in oxygen availability as they progress through their life cycle. Metacercariae, and other free-living stages, readily obtain oxygen from the environment. However, once infections become established and flukes migrate within host tissues, they begin to encounter environmental hypoxia (72, 73), which coincides with a switch from aerobic energy metabolism (TCA cycle) to anaerobic malate dismutation (74). Our transcriptome analysis indicates that *C. daubneyi* follows this metabolic strategy with TCA cycle members expressed most highly by the NEJ stage and becoming gradually downregulated as the parasite matures to the adult stage. Coincident with the steady decline of the TCA pathway is the upregulation of members of the malate dismutation pathway with parasite development. Such gradual change of metabolic pathways could suggest the existence of multiple populations of mitochondria in *C. daubneyi* each with different respiratory activities/tissue locations as have been described for the lung fluke, *Paragonimus westermani* (75). This would provide the metabolic plasticity required as *C. daubneyi* moves from aerobic conditions within the duodenal submucosa toward an increasingly anaerobic environment (*i.e.*, the rumen) and ensure that efficient ATP production is maintained to support the enormous energy demands of egg production that begins after this migration. While the biochemistry of *C. daubneyi* mitochondria require further investigation, Huson *et al*. (54) did report the production of propionate (a major excretory product of malate dismutation) by adult *C. daubneyi* during *in vitro* culture, which supports the current transcriptome data.

After excystment in the duodenum, NEJs must respond rapidly to adapt to their new environment and to counter inevitable attack by the host immune system (41). At the transcriptional level, *C. daubneyi* orthologues of trematode proteins with known immunomodulatory function including kunitz-type inhibitor, peroxiredoxin, FaBPs, venom allergen–like proteins, and GSTs were largely upregulated in the NEJ stage. In *F. hepatica*, and other trematodes, these proteins act, *via* a variety of mechanisms, to establish a Th2 immune environment that is permissive to parasite survival and reproduction (76, 77, 78, 79, 80, 81). While the host immune response to *C. daubneyi* infection (and to what extent the parasite can manipulate this) has yet to be characterized in detail, the NEJ stage is clearly equipped with sufficient armory to avoid the immune response and establish initial infection in the duodenal mucosa. However, field observations and experimental studies indicate that previous exposure of livestock to rumen fluke provides protection (routinely >99%) against massive infections that would typically cause acute (and often fatal) paramphistomosis (7, 82, 83, 84). Critically, these studies showed that immunization with adult worms (delivered orally to establish infection directly in the rumen) was not effective and that protective immunity requires exposure of the duodenum to NEJ-derived antigen (7). This is in stark contrast to liver fluke, where there is limited evidence of natural acquired immunity in cattle and less so in sheep (85). As a result, attempts to vaccinate livestock against liver fluke have been largely unsuccessful, despite a considerable research effort (86). Thus, rumen fluke may be more amenable to vaccinal control than liver fluke infection, and our proteomics analysis of NEJ secretions has unveiled a range of early-stage molecules that represent potential candidates for vaccine development.

The single most abundant protein found in the adult fluke secretome was CdHDM-3. This represents one of the three isoforms of the HDMs, a family of trematode-specific proteins with diverse immunomodulatory functions; *F. hepatica* HDM-1 has been shown to protect mice against lipopolysaccharide induced inflammation by significantly reducing the release of inflammatory cytokines from macrophages (38) and to suppress antigen processing and presentation in macrophages *via* inhibition of lysosomal vacuolar ATPase (39). This is the first time HDMs have been reported in suborder Pronocephalata and the first time it has been identified in the secretions of *C. daubneyi*, presumably not being resolved on the 2-DE used by Huson *et al*. (54) because of its size (∼6 kDa). While the function of HDMs during *C. daubneyi* infection is unknown, two of the isoforms were predicted to possess a C-terminal amphipathic helix, which is the key functional determinant of the lipopolysaccharide-binding/immunomodulatory properties of HDMs from other trematodes (38, 39, 40). Thus, *C. daubneyi* may use HDMs to simultaneously maintain a Th2 immune environment and to protect against pathogen-associated molecular patterns in the microbiome-rich rumen—both of which would serve to sustain chronic infections that can persist in livestock for many years (7).

In trematodes, cathepsin-like cysteine peptidases play central roles in virulence, infection, tissue migration, and modulation of host immune responses (44). While cathepsin B peptidases are expressed by most species, there is clear divergence in the use of other types of cathepsins: *Fasciola* spp. and *Schistosoma* spp. predominantly express cathepsins L, whereas cathepsins F are the major peptidases expressed by *Clonorchis* spp., *Paragonimus* spp., and *Opisthorchis* spp (43). Our transcriptome and phylogenetic analysis showed that the *C. daubneyi* cathepsin B family has undergone considerable expansion and has diverged into six clades with varying S2 subsite residues. Because the composition and arrangement of amino acids that create the S2 subsite within the cathepsin active site determine the specificity of the enzyme (51, 87), it is likely that the *C. daubneyi* cathepsin B clade members represent a repertoire of enzymes with overlapping/complementary substrate specificities that are capable of degrading a variety of host macromolecules they encounter as they develop and migrate within the host. In support of this, the clade members were found, at both the transcript and protein levels, to be developmentally regulated with clades 1, 3A, and 5 expressed/secreted predominantly by the NEJs/immature stage and clades 2, 3B, 4, and 6 expressed/secreted by newly migrated/adult flukes.

*C. daubneyi* was also found to have a large aspartic peptidase family that could be segregated into distinct clades largely expressed by the adult flukes. Despite this, aspartic peptidases were not particularly abundant in the secretome. This mirrors *O. viverrini* which, despite having 27 aspartic peptidase genes, secrete only low levels of the enzyme (58, 88). Although it is not currently possible to predict the active site composition of aspartic peptidases, it is likely that, given their expansion and clear developmental regulation, they too will have varying substrate preferences. In blood-feeding helminths, aspartic peptidases usually form part of a multienzyme cascade, with endopeptidase and exopeptidase activity, to degrade host hemoglobin (89). While the source of nutrition for *C. daubneyi* has yet to be confirmed (*e.g.*, host tissue, rumen fluid, rumen microorganisms), such an enzyme cascade is possible because other digestive enzymes including aminopeptidases, carboxypeptidases, and chymotrypsin-like peptidases were all found in the secretome. This would be in contrast to *F. hepatica,* which relies solely on an expanded repertoire of cathepsin L peptidases, which comprise >80% of the total soluble protein secreted by adult fluke, to degrade hemoglobin (43). Here, we found only two transcripts encoding cathepsin L (and one encoding cathepsin F), which were secreted by NEJs in low abundance. While these retain key active site residues, indicating that they will form functional enzymes, they lack conserved asparagines at the junction of the prosegment and mature domain. This suggests that they are not *trans-*activated to the mature enzyme by asparaginyl endopeptidase (legumain; although these were also found in *C. daubneyi* secretions) but could be activated by cathepsin B as reported for *O. viverrini* cathepsin F (90).

Owing to their abundance in liver fluke secretions, cathepsin L is the target antigen for most coproantigen-based ELISA tests developed for *F. hepatica* infection (91, 92). The MM3 mAb used for antigen capture in *F. hepatica* coproantigen assays does not cross-react with rumen fluke antigens either on histological sections or by ELISA (93). Our secretome analysis has now confirmed that this is because *C. daubneyi* secrete very little cathepsin L, mostly from the NEJ stage. Thus, while specific tests for fasciolosis are available (which would be of particular value in areas where paramphistomosis also occurs, either singly or as coinfections with *F. hepatica*), there are currently no such tests for rumen fluke infection. As a result of the significant differences in the profile of molecules secreted by both species, we investigated the diagnostic value of *C. daubneyi* E/S antigens in a coproantigen-based ELISA. The results demonstrate that nanogram amounts of *C. daubneyi* E/S antigen could be captured from fecal extracts with sufficient sensitivity to distinguish between naturally infected and uninfected cattle. Importantly, the anti–CdE/S serum did not cross-react with somatic or secretory antigens from a range of helminth species that commonly share the same ruminant hosts as *C. daubneyi.* The use of this coproantigen ELISA would offer a noninvasive means of diagnosing current rumen fluke infection (antigens only persist for the duration of infection) and could also be used for the detection of drug resistance (94); because the current treatment is reliant on a single drug (oxyclozanide), this seems inevitable (1).

In this report, we have generated the first transcriptome resources for multiple intramammalian life-cycle stages of *C. daubneyi*. By integrating this with secretome analysis of the most clinically relevant stages, we provide a comprehensive, and dynamic, overview of initial infection in the duodenum, migration within the gastrointestinal tract, and final maturation upon arrival in the rumen. In particular, our analysis of the molecules expressed/secreted by the NEJ stage provides a valuable framework for studies aimed at better understanding infectivity and how drugs or vaccines could be developed to prevent infection and/or limit damaging intestinal pathology associated with acute paramphistomosis. Because prepatent rumen fluke infections are the primary cause of clinical disease (4), our NEJ secretome data have yielded a range of putative early-stage antigens that could allow timely diagnosis and treatment for this emerging infection.

## Data availability

The transcriptome datasets supporting the conclusions of this article are available in the European Nucleotide Archive (ENA) repository under accession number PRJEB28150, http://www.ebi.ac.uk/ena/data/view/PRJEB28150. The MS proteomics data have been deposited to the ProteomeXchange Consortium *via* the PRIDE partner repository with the dataset identifier PXD014550 and 10.6019/PXD014550.

## Supplemental data

This article contains supplemental data.

## Conflict of interest

The authors declare no competing interests.
