# Supplementary material for: Transcriptome and Secretome Analysis of Intra-Mammalian Life-Stages of Calicophoron daubneyi Reveals Adaptation to a Unique Host Environment
Source: Mol Cell Proteomics. 2021 Feb 11;20:100055. doi: 10.1074/mcp.RA120.002175 (PMC7973311; doi:10.1074/mcp.RA120.002175)
Supplement: Figure and Table legend [file mmc1.docx]

**Supplementary files**

**Supplementary Table S1:** Annotation of the top 10% DE transcript sequences.

**Supplementary Table S2:** Number of differentially-expressed (DE) transcripts identified from analysis of Illumina RNA-seq data. The first row shows the total number of DE transcripts and the subsequent two rows show how many of these were up- and down-regulated respectively. NM, newly-migrated fluke; I, immature fluke; NEJ, newly-excysted juvenile; A, adult fluke.

**Supplementary Table S3:** Identification of *C. daubneyi* NEJ and adult E/S proteins by LC-MS/MS.

**Supplementary Figure S1.** Principal component analysis (PCA) of the *C. daubneyi* RNAseq data.

The plot shows there is consistent behaviour (clustering) across the three biological replicates of each life-cycle stage. Triplicate samples for each of the life-cycle stages are shown, NEJ (blue); immature flukes (green); newly-migrated flukes (red); adult flukes (black).

**Supplementary Figure S2.** Graphical representation of transcript expression for catalytic enzymes of glycolysis/gluconeogenesis (A) TCA cycle (B) and malate dismutation (C) pathways presented as heatmaps for newly-excysted juveniles (NEJ), immature larvae, newly-migrated (NM) and adult *C. daubneyi*. Relative expression is indicated by a blue to red scale bar portraying low to high levels of expression respectively.

**Supplementary Figure S3.** Sequence analysis of the helminth defence molecules (HDMs) expressed by *C. daubneyi.*

(A) A bootstrapped (1000 trials) neighbour-joining phylogenetic tree showing the evolutionary relationship of HDM amino acid sequences from various trematode species. Numbers represent bootstrap values for a particular node, and values greater than 50% are shown. The tree is rooted to the hydrophobic ligand-binding protein (Uniprot accession Q9GPP5) from the tapeworn *Moniezia expansa*. (B) Helical wheel analysis shows that the C-terminal hydrophobic regions form distinct amphipathic helices in FhHDM-1, CdHDM-2 and CdHDM-3 but not in CdHDM-1. (C) Primary sequence alignment of FhHDM-1 with *C. daubneyi* HDMs 1-3. The C-terminal hydrophobic region that forms the amphipathic helix is coloured red in FhHDM-1.

**Supplementary Figure S4.** Phylogenetic relationships of the *C. daubneyi* aspartic peptidase family and their developmental expression patterns.

(A) Bootstrapped (1000 trials) neighbour-joining phylogenetic tree showing the evolutionary relationship of *C. daubneyi* aspartic peptidase amino acid sequences. Numbers represent bootstrap values (given as percentages) for a particular node and values greater than 50% are shown. The tree is rooted to *Arabidopsis thaliana* aspartic peptidase (Uniprot accession Q9M9A8). (B) The TPM values of transcripts encoding the aspartic peptidase clade members were represented as a heatmap to visualise their expression patterns across the intra-mammalian life-cycle stages of *C. daubneyi*. Relative expression is shown by a red to green scale depicting low to high levels of expression, respectively.
