# Supplementary material for: Transcriptome and Secretome Analysis of Intra-Mammalian Life-Stages of Calicophoron daubneyi Reveals Adaptation to a Unique Host Environment
Source: Mol Cell Proteomics. 2021 Feb 11;20:100055. doi: 10.1074/mcp.RA120.002175 (PMC7973311; doi:10.1074/mcp.RA120.002175)
Supplement: Table S2 [file mmc3.docx]

**Supplementary Table S2:** Number of differentially-expressed (DE) transcripts identified from analysis of Illumina RNA-seq data. The first row shows the total number of DE transcripts and the subsequent two rows show how many of these were up- and down-regulated respectively. NM, newly-migrated fluke; I, immature fluke; NEJ, newly-excysted juvenile; A, adult fluke.

|  | NM vs A | I vs A | NEJ vs A | I vs NM | NEJ vs NM | NEJ vs I |
| --- | --- | --- | --- | --- | --- | --- |
| Number of contigs differentially expressed between developmental stages | 11367 | 13606 | 14838 | 7778 | 12294 | 11821 |
| Number up-regulated in ‘A’ vs ‘B’ comparison | 5224 | 6064 | 6751 | 3585 | 5946 | 5882 |
| Number down-regulated in ‘A’ vs ‘B’ comparison | 6143 | 7542 | 8087 | 4193 | 6348 | 5939 |
