# Supplementary figures and images for: Transcriptome and Secretome Analysis of Intra-Mammalian Life-Stages of Calicophoron daubneyi Reveals Adaptation to a Unique Host Environment
Source: Mol Cell Proteomics. 2021 Feb 11;20:100055. doi: 10.1074/mcp.RA120.002175 (PMC7973311; doi:10.1074/mcp.RA120.002175)

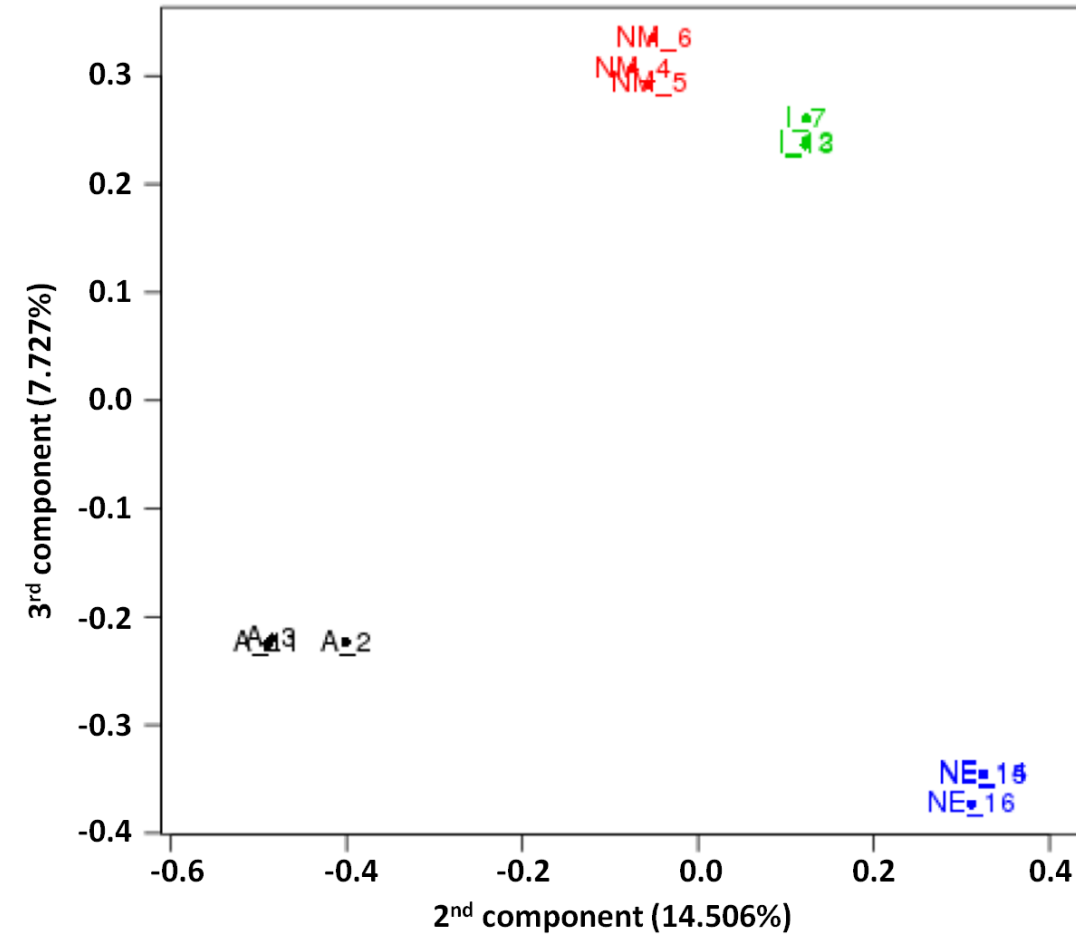

Supplement: Figure S1 [file mmc5.pdf]

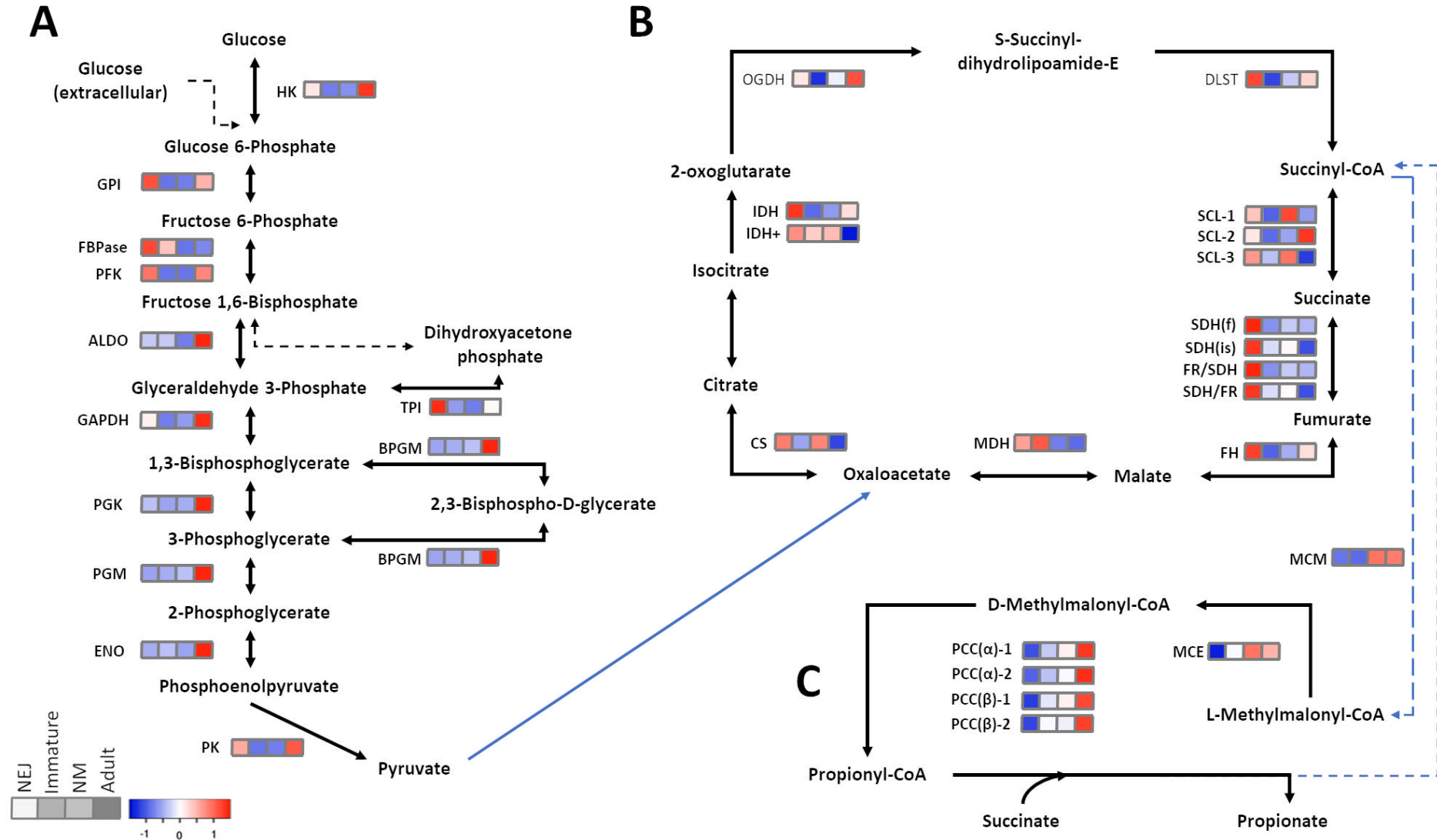

Supplement: Figure S2 [file mmc6.pdf]

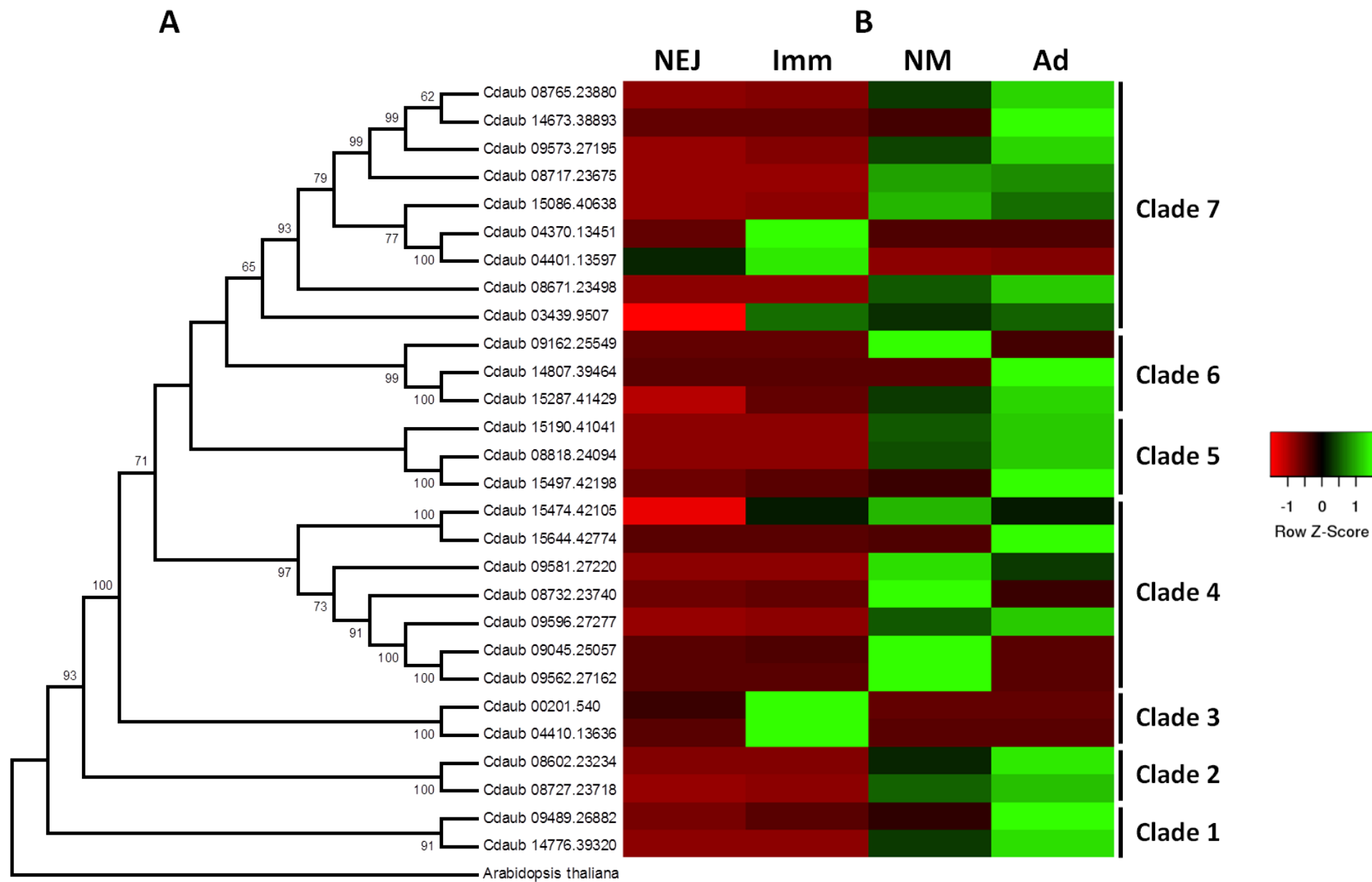

Supplement: Figure S4 [file mmc8.pdf]
