# Supplementary material for: Transcriptome and Secretome Analysis of Intra-Mammalian Life-Stages of Calicophoron daubneyi Reveals Adaptation to a Unique Host Environment
Source: Mol Cell Proteomics. 2021 Feb 11;20:100055. doi: 10.1074/mcp.RA120.002175 (PMC7973311; doi:10.1074/mcp.RA120.002175)
Supplement: Figure S3 [file mmc7.pdf]

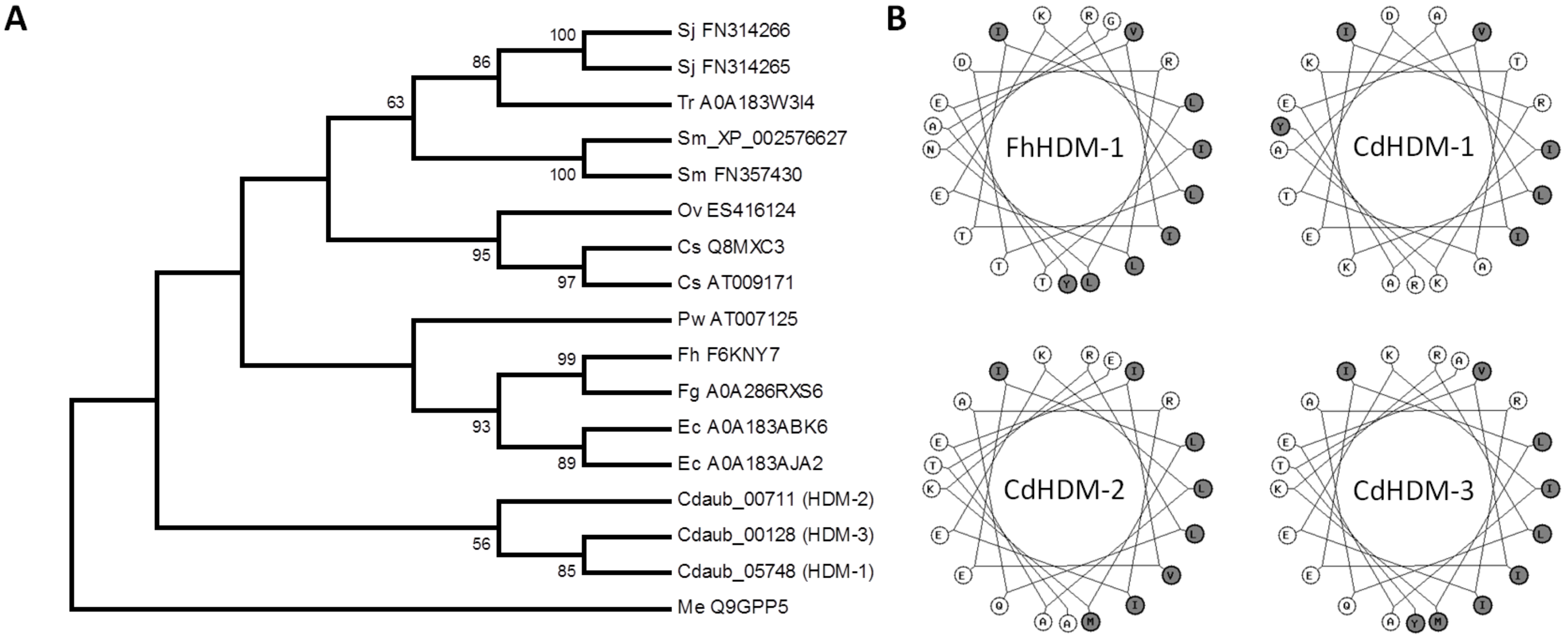

C

```
FhHDM-1    RPSEESREKLRESGRKMVKALRDAVTKAYEKARDRAMAYLAKDNLGEKITEVITILLNRLTDRLEKYAGN--   70
CdHDM-1    RPNEQTREKLRESGTKLLTAIRHAIEKIRAKTQEKVEAYMEKDGLGEKIAEVIEIRKAALKKTATDRY----   68
CdHDM-2    RPDEATRARLRESGTKLWKAVGEACEKIRAKIRAKVDAYFEKDGLGEKLAEIVEILMKRLQARIEKATEKIE   72
CdHDM-3    RPNEQTREKLRESGTKLLTAIRHAIEKIRAKTQEKVEAYMEKDGLGEKIAEVIEILMKRLQARI-EKYTAEH   71
           **.* :* :***** *: .*: .*  *   * : :. **: **.****::*:: *    *     .
```
